# Antibacterial Activity of Allicin-Inspired Disulfide Derivatives against *Xanthomonas* *axonopodis* pv. *citri*

**DOI:** 10.3390/ijms231911947

**Published:** 2022-10-08

**Authors:** Mei Zhu, Yan Li, Xuesha Long, Congyu Wang, Guiping Ouyang, Zhenchao Wang

**Affiliations:** 1 Center for Research and Development of Fine Chemicals, Guizhou University, Guiyang 550025, China; 2 School of Pharmacy, Guizhou University, Guiyang 550025, China

**Keywords:** Disulfides, *Xanthomonas* *axonopodis* pv. *citri*, quantitative proteomics, MEP pathway

## Abstract

*Xanthomonas axonopodis* pv. *citri (Xac)* belongs to the Gram-negative species, causing citrus canker that seriously affects the fruit yield and quality of many rutaceae plants. Herein, we found that compound 2-(butyldisulfanyl) quinazolin-4(3*H*)-one exhibited remarkable anti-*Xac* activity in vitro with a half effective concentration (EC_50_) of 2.6 μg/mL, while the positive controls thiodiazole-copper with 57 μg/mL and bismerthiazol with 68 μg/mL and this compound showed great anti-citrus canker activity in vivo. This active compound also was confirmed to reduce biofilm formation, increase the level of reactive oxygen species, damage the morphological structure of the bacteria, and cause bacterial death. Proteomics and RT-qPCR analysis results indicated that this compound down-regulated the expression of enzymes in the MEP (2-methyl-D-erythritol 4-phosphate) pathway and might achieve destructive ability of *Xac*. Overall, this study indicates that such derivatives could be a promising scaffold to develop novel bactericides to control citrus canker.

## 1. Introduction

Citrus canker, caused by *Xanthomonas axonopodis* pv. *citri* (*Xac*), is a destructive bacterial disease which is an extreme constraint on citrus production. The bacteria belong to the Gram-negative species and seriously threaten many rutaceae plants [1,2,3]. At present, most of the effective bactericides used for this disease are copper agents, which achieve bactericidal activity by destroying the integrity of the membrane, combining with proteins and DNA in the bacteria, and inducing the production of free radicals, etc. [4,5]. To achieve adequate protection, multiple applications of a copper-agent are necessary, leading to soil and water pollution due to the undesirable degradation of copper, and copper resistant pathogens have emerged during long-term use [3,6]. Thus, it is necessary to find an efficient green bactericide to prevent or control citrus canker.

Isoprenoids include a diverse family of over 35,000 natural products, and their biosynthesis begins with the repeated condensation of a C5 precursor, isopentenyl pyrophosphate (IPP) [7]. Isoprenoid precursors are prepared by the mevalonate (MVA) and 2-methyl-D-erythritol 4-phosphate (MEP) pathways [8]. Previous research showed that the MEP pathway for the biosynthesis of terpenoids is essential for the survival of most bacteria, including human- and plant- pathogens, but is absent in mammals. Thus, exploring inhibitors of the MEP pathway could be a new therapeutic way to treat plant bacterial diseases, and each of the seven enzymes in the MEP pathway might represent a target that could yield a new antibacterial class [8,9,10,11].

Natural products usually have unique pharmacophore patterns and have become the most important source of inspiration to find antibacterial active compounds. Quinazolinone alkaloids and their derivatives have shown superb bioactivities, such as antibacterial, antiviral, and anti-inflammatory activities [12,13,14,15]. As another class of natural products, allicin is a reactive sulfur species that has been used as a natural bactericide for a long time. It has a wide range of cellular targets and is a concentration-dependent biocide [16,17,18]. Quinazolin-4(3*H*)-one derivatives showed good biological activity after introducing a thioether structure into position 2 [14,19,20]. This served as the inspiration for our compound structure design. Surprisingly, after splicing these two active structures, many target compounds containing disulfide bonds with high inhibitory activity against *Xac* were obtained.

## 2. Results

### 2.1. In Vitro Antibacterial Activity of Target Compounds

First, 2-thioxo-2,3-dihydroquinazolin-4(1*H*)-one (intermediate 1) was prepared from 2-aminobenzamide and carbon disulfide through a cyclization reaction, and various hypochlorothioite (intermediate 2) were prepared from thiol and sulfuryl chloride through substitution reactions. Then, target compounds 1−20 were prepared from intermediates 1 and 2 in ethyl ether through a substitution reaction, with yields ranging from 1.0% to 86%.

**Figure 1:**
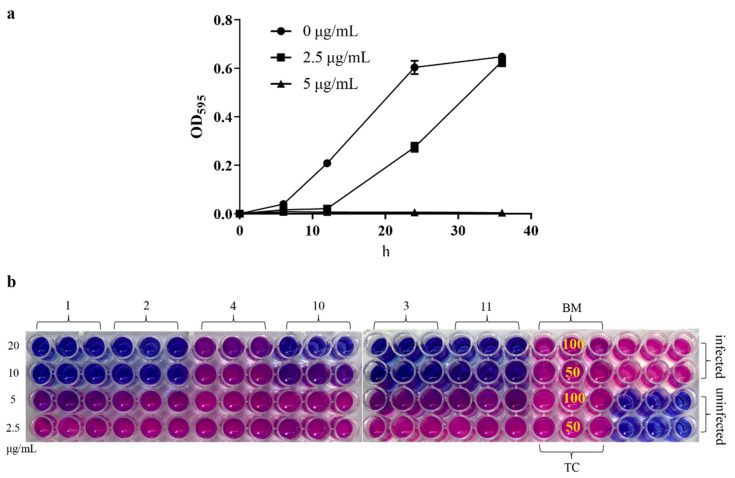
(**a**) The growth curve of *Xac* under the action of compound 1; the test concentrations were 5.0 and 2.5 μg/mL, and the absorbance was measured at 595 nm at 0, 6, 12, 24 and 36 h, respectively. (**b**) Resazurin blue assay, the test concentrations of target compound were 20, 10, 5.0, and 2.5 μg/mL, and the test concentrations of BM and TC were 100 and 50 μg/mL for the compound-treated groups. Pink indicates bacterial growth and blue means no bacterial growth (the corresponding concentration is the MIC value).

In order to evaluate the anti-*Xac* activity of the target compounds in vitro, a turbidimeter test and resazurin blue assay were used. The results of the turbidimeter test (Table 1 and Table 2) showed that most target compounds exhibited significant inhibitory effects against *Xac* in vitro. The EC_50_ values of compound 1−4, 10, and 11 were less than 10 μg/mL, which were superior to that of bismerthiazol (BM, 68 μg/mL) and thiodiazole copper (TC, 57 μg/mL), among which compound 1 (2-(butyldisulfanyl) quinazolin-4(3*H*)-one) showed the best activity (2.6 μg/mL). Then, from the growth curve of *Xac* (Figure 1a), compound 1 could effectively inhibit the growth of bacteria at 5.0 μg/mL (about 2×EC_50_). According to the results of the resazurin blue assay (Figure 1b), compound 1 also displayed great inhibitory activity against *Xac* in vitro with a MIC (minimum inhibitory concentration) value of about 10 μg/mL, compared with the positive control BM and TC, which were more than 100 μg/mL.

### 2.2. Antibacterial Activity Assay in Leaf of Navel Orange

**Figure 2:**
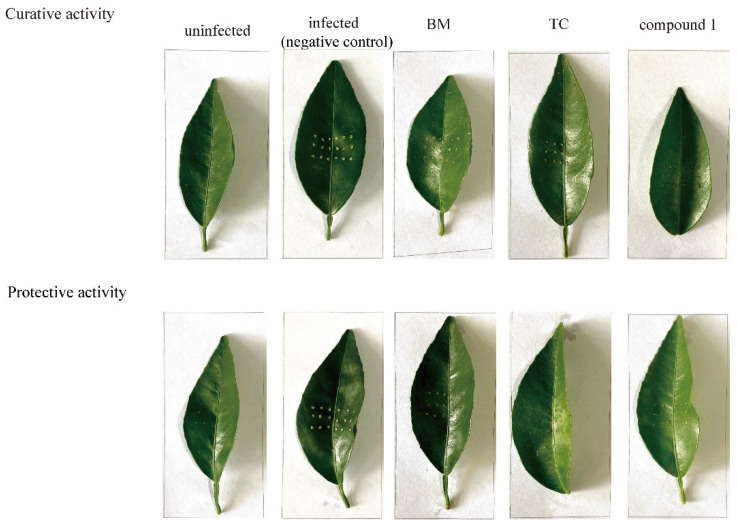
Curative and protective activity of compound 1 against citrus canker in fully expanded young citrus leaves of navel orange; the test concentration was 200 μg/mL. The sample was placed in a 28 °C constant temperature incubator and absorbent filter paper was used for moisturizing. The spots on the inoculated leaves were observed after 7 days.

Compound 1 showed good antibacterial activity against *Xac* in vitro. The anti- citrus canker activity of compound 1 on leaves was determined to evaluate its activity in vivo. After 7 days of incubation at 28 °C, no yellow spots on the uninfected leaves, but obvious crater-like swell spots appeared on the infected citrus leaf surface (negative control). In contrast, spots weakened compared with the negative control after compound 1, BM, and TC treatment (200 μg/mL). For the curative activity, the surface of leaf treated with compound 1 showed smaller spots (slightly yellow) than the positive drug-treated leaves, indicating that the curative activity of compound 1 was better than that of the positive control (BM and TC). Compound 1 showed similar protective activity with TC (all showed small volcanic spots) and was better than BM (Figure 2).

### 2.3. Effects of Compound 1 on the Formation of Bacterial Biofilm, Apoptosis, and Morphology

**Figure 3:**
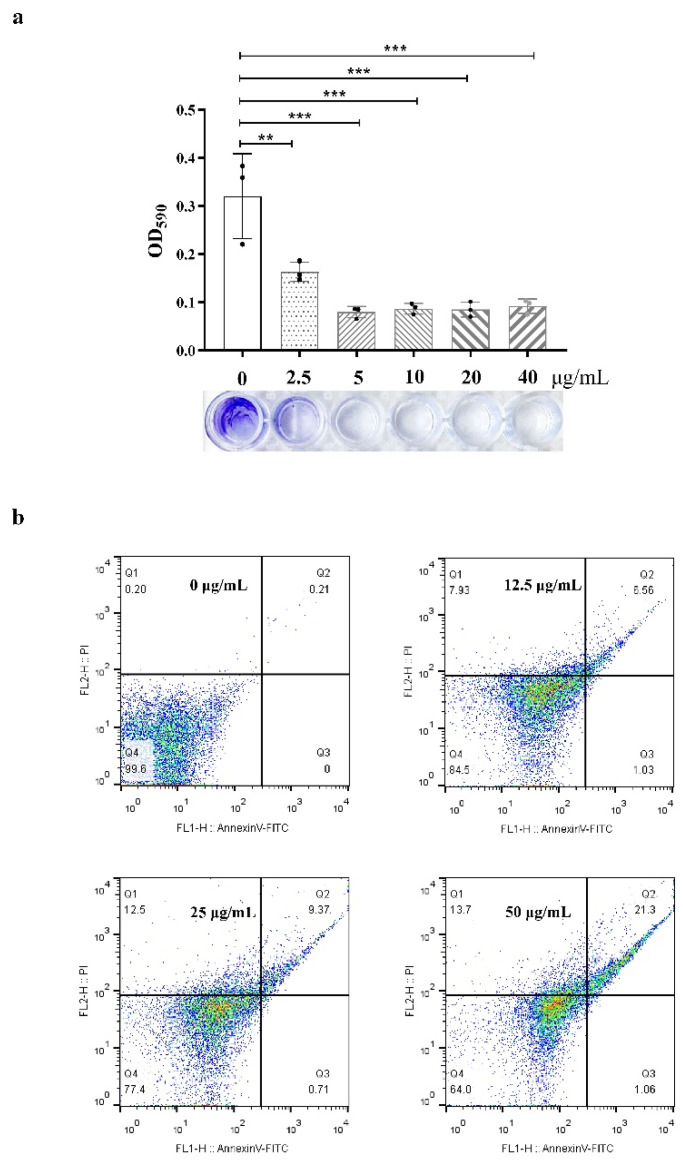
(**a**), Biofilm formation (crystal violet staining); the test concentrations were 40, 20, 10, 5.0, and 2.5 μg/mL and the absorbance value of crystal violet was measured at 590 nm. ** for *p* < 0.01, *** *p* < 0.001, One-way ANOVA followed by Dunnett’s multiple comparisons test was performed using GraphPad Prism. (**b**), Apoptosis detection (PI and AnnexinV-FITC staining); the test concentrations were 50, 25, and 12.5 μg/mL. Q1 means necrosis cell, Q2 means late apoptosis, Q3 means early apoptosis, and Q4 means live bacteria.

To further understand the effects of compound 1 on bacterial pathogenicity, the formation of bacterial biofilms, changes in bacterial morphology, and apoptosis were determined. From the results of the biofilm formation assay, with the increase of test concentration, crystal violet staining on 96-well plates decreased obviously (Figure 3a) when the concentration was 2.5 μg/mL. Additionally, the absorbance value of crystal violet at 590 nm decreased to half, which means that the formation of bacterial biofilm was significantly reduced under the action of compound 1. PI and FITC staining were used to determine bacterial viability; the results (Figure 3b) showed that the ratio of dead bacteria was concentration-dependent, i.e., when the concentration was 12.5 μg/mL, the ratio was 15%, and for 50 μg/mL, the ratio was 36%. However, there does not seem to be a substantial increase in FITC fluorescence when PI fluorescence is absent, as the percentage of early apoptosis cells barely increased.

**Figure 4:**
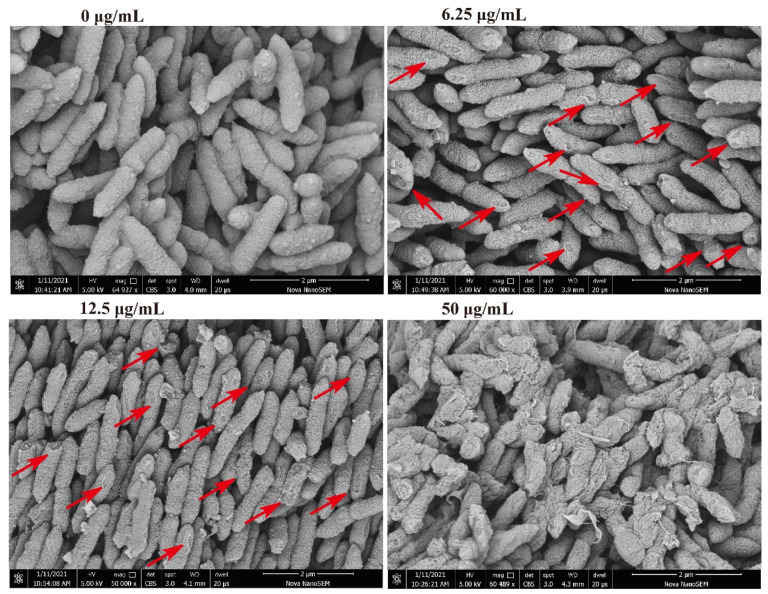
Changes of morphological structure of *Xac* under the action of compound 1. The test concentrations were 50, 12.5, and 6.25 μg/mL. Red arrows point to areas with obvious structural damage.

Regarding the bacterial morphology, as the concentration of compound 1 increased, part-broken, ruptured, and even fragmentation occurred. At 6.25 μg/mL, the middle and both ends of the bacteria began to show some damage, while at 50 μg/mL, the bacterium demonstrated significant damage and many fragmented structures (Figure 4), consistent with the results of apoptosis detection. As such, this compound was shown to destroy the bacterial wall structure, leading to cell death. The SEM results explain the reason that the proportion of dead cells increased with increasing concentration in the apoptosis detection; however, there did not seem to be a significant increase in FITC fluorescence in the absence of concomitant PI. As such, this may not have been due to programmed cell death caused by phosphatidylserine (PS); it is more likely that the damage to the cell structure allowed the PI dye enter the bacteria.

### 2.4. Quantitative Proteomics Results and RT-qPCR Analysis

**Scheme 1:**
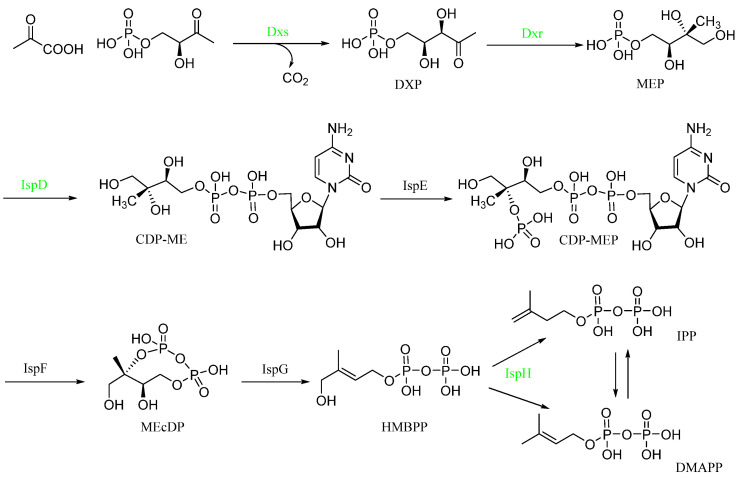
KEGG enrichment analysis of the MEP pathway (converts the initial substrates pyruvate and glyceraldehyde 3-phosphate to IPP or DMAPP through a series of seven enzymatic reactions). Green means down-regulated enzyme from the Proteomics results.

**Figure 5:**
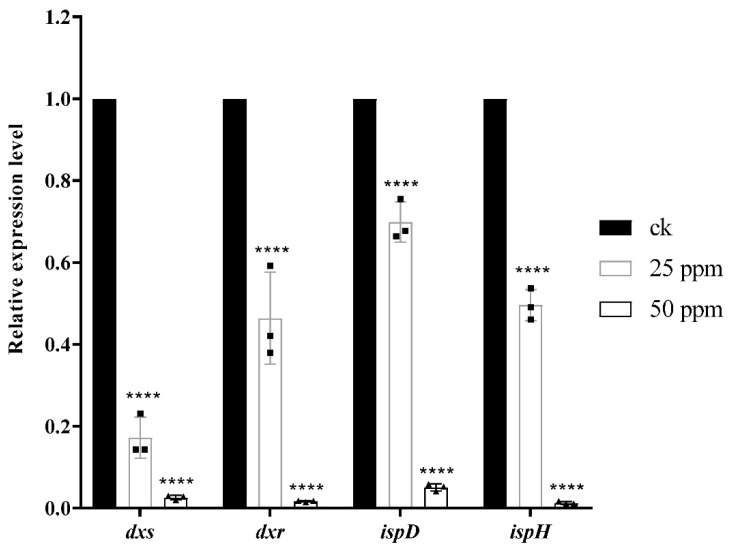
Analysis of the relative expression of related genes (*dxs*, *dxr*, *ispD*, *ispH*) through RT-qPCR (2^−∆∆Ct^), ****p < 0.0001, One-way ANOVA followed by Dunnett’s multiple comparisons test was performed using GraphPad Prism.

Compound 1 caused damage to the bacterial structure. To further speculate about the potential anti-*Xac* mechanism of compound 1, the differential proteins caused by the action of this compound were determined through Proteomics. The results (Figure S1) comprised 296,169 spectrums. Additionally, 2076 proteins were identified, and 1958 proteins were quantified. Compared with the negative control, after treatment with compound 1, the number of up-regulated proteins was 275, and the number of down-regulated proteins was 106. Through the enrichment and analysis of the KEGG pathway, it was found that four catalytic enzymes were down-regulated in the biosynthesis pathway of the terpenoid backbone (MEP pathway), namely, Dxs (1-deoxy-D-xylulose 5-phosphate synthase), Dxr (1-deoxy-D-xylulose-5-phosphate reductoisomerase), IspD (4-diphosphocytidyl 2-C-methyl-D-erythritol cytidyltransferase), and IspH (4-hydroxy-3-methylbut-2-en-1-yl diphosphate reductase) (Scheme 1), which were encoded by *dxs*, *dxr*, *ispD*, *ispH* gene, respectively. The PCR assay (Table 3) results showed that the relative expression levels of these four genes became increasingly down-regulated as the concentration increased; especially at 50 μg/mL, the expression level of the four genes decreased significantly compared with the negative control (Figure 5).

## 3. Discussion

*Xanthomonas* causes many plant diseases, such as leaf spot, leaf blight, wilt, and ulcer. Specifically, *Xanthomonas axonopodis* pv. *citri* causes citrus canker, a condition which is usually avoided by strict control and quarantine. However, once it occurs, it is difficult to eradicate, as few effective bactericides are available, and most are copper agents with some shortcomings. Thus, it is necessary to find an effective green bactericide that can replace copper-based agents to control citrus canker.

In this study, a series of compounds with anti-*Xac* activity were synthesized and evaluated, among which compound 1 (2-(butyldisulfanyl) quinazolin-4(3*H*)-one) showed the best activity (EC_50_ = 2.6 μg/mL) against *Xac* in vitro. In general, when the R group of the target compounds was a fatty chain, the anti-*Xac* activity was better than with an aromatic ring substitution. For fatty groups, the activity decreased significantly when the number of carbon atoms increased, and the activity of the linear chain was better than that of the ring group. The inhibitory rate was 92.91% at 100 μg/mL for hexyl substitution (compound 7) and 37.26% for cyclohexyl (compound 8) from Table 1. The phenyl (compound 11) had great anti-*Xac* activity with the value of EC_50_ was 2.7 μg/mL; however, the activity was poor when there were substituents on the benzene ring, such as electron donor groups (methyl and methoxy). When the substitution changed to a halogen, the activity improved; fluorine was better than chlorine and bromine in the para position. The inhibition rates were 87.83%, 43.62%, and 38.93% at 100 μg/mL, respectively (Table 1). In addition, when fluorine substitution occurred at position 3 (compound 20), it showed great antibacterial activity, with an EC_50_ of 12 μg/mL (Table 2). When substitution occurred on the benzene ring, the activity decreased, and the activity of halogen substitution was better than that of electron donor substitution. At the same time, the activity of fluorine was better than that of chlorine and bromine. This may have been due to the fact that the fluorine atom is small. Subsequently, compound 1 was deemed to be the most active anti-citrus canker compound; the disease spots showed that this compound has great curative and protective activities, compared with copper-agent TC.

Next, we address the following question: does compound 1 achieve its anti-*Xac* activity by affecting the bacterial integrity, metabolism or virulence? Firstly, starting with the appearance, we determined the effects of compound 1 on biofilm formation and cell structure and determined whether the compound causes bacterial death. The results showed that biofilm formation decreased under the action of compound 1, which may indicate that the pathogenicity of *Xac* decreased; this was consistent with the anti-citrus canker activity results. An SEM image (Figure 4) showed that the bacterial cell wall was seriously damaged. As such, the survival status of bacteria could then be determined by fluorescent dye (Figure 3b). FITC- labeled Annexin V, coupled with propidium iodide (PI) for determination of cell viability, can be utilized for quantification by flow cytometry of exposed PS in a dying bacterium [21]. The results showed that compound 1 destroyed the bacterial wall structure, leading to death. Was the cause of cell damage due to the interaction between compound 1 and the cell wall or did it affect the biosynthesis of the bacterial wall through other mechanisms? Secondly, the differential proteins between the compound 1- treated group and the untreated group were determined by proteomics. An enrichment analysis of differential proteins showed that the expression of four enzymes (Dxs, Dxr, IspD, and IspH) in the MEP pathway were downregulated, and the PCR results (Figure 5) also verified that the expression of the corresponding genes was downregulated. Previous reports indicated that the MEP pathway for the biosynthesis of terpenoids is essential for the survival of bacteria and that it involves the biosynthesis of the cell wall [22,23,24]. Thus, this may be a possible mechanism for this compound to cause cell wall damage and death. In addition, isoprenoids are involved in numerous other essential cellular processes, such as cellular respiration, cell signaling, and oxidation-reduction [25]. The reactive oxygen species (ROS) levels also increased significantly under the action of compound 1 (Figure S2). This effect in *Xac* may have been due to the inhibition of isoprene synthesis [26]. In a word, the specific mode of action is still unclear. Some of the anti-*Xac* effects may have been achieved through the inhibition of the MEP pathway. However, it is still necessary to determine which protein directly interacts with this compound to cause the down-regulation of enzymes in that pathway.

## 4. Materials and Methods

### 4.1. Equipment and Materials

Unless otherwise stated, all reagents and solvents were obtained commercially and used without further purification. NMR spectra were acquired with a JEOL-ECX instrument at room temperature using DMSO-*d6* or CDCl_3_ as a solution and TMS as an internal standard. Thermo Scientific Q Exactive was used to record high-resolution mass spectrometry (HRMS) spectra. BD FACSCalibur flow cytometry was used to detect the fluorescence intensity. Nova Nano SEM 450 was used to observe the morphology of bacteria. In this study, data analyses were conducted using GraphPad Prism 7.04.

### 4.2. Synthetic Procedure

The synthetic procedure of the title compounds was performed according to a previously reported method [27,28]. The chemical structures of the target compounds were characterized via ^1^H NMR spectroscopy, ^13^C NMR spectroscopy, and high-resolution mass spectrometry (HRMS). Detailed characterization data are presented in the Supporting Information (S1).

### 4.3. Bacterial Strain and Culture Conditions

*Xanthomonas axonopodis* pv. *citri* wild-type sensitive strain *Xac*306 was used. Static culture on nutrient agar (NA) containing 5.0 g/L of peptone, 10 g/L of sucrose, 1.0 g/L of yeast powder, 3.0 g/L of beef extract, and 15 g/L of agar powder was used, and the pH was adjusted to 7.0 with sodium hydroxide. The nutrient broth (NB) contained the same ingredients except for agar, which was used for our experiments and for shock culture tests at 28 °C, 250 rpm. In this study, unless otherwise specified, all experimental bacterial solutions were obtained by culturing on NA medium for 48 h and then transferring to NB medium for shocking culture. The growth state was determined by the absorbance value at 595 nm. The title compound, bismerthiazol (BM, 90%), and thiodiazole copper (TC, 20% suspending agent) were dissolved in dimethyl sulfoxide (DMSO).

### 4.4. In Vitro Antibacterial Activity of Title Compounds

All title compounds were evaluated for their antibacterial activities against *Xanthomonas axonopodis* pv. *citri* (*Xac*) by the turbidimeter test [29,30] and resazurin blue assay [10], as described elsewhere. For the turbidimeter test, DMSO was added to sterile distilled water for the negative control, while BM and TC served as the positive control. Then, 40 μL bacterial solutions (NB solution, OD_595_ ≈ 0.6) were added to a test tube containing 4 mL of the mixed solution of NB and test compound. This was incubated at 28 °C and shaken continuously at 250 rpm for 12−24 h until the bacteria (negative control) grew to the logarithmic growth phase. The growth of the bacteria was monitored on a microplate reader by measuring the absorbance value at 595 nm. The inhibition rates I (%) were calculated by the formula I (%) = (C-T)/C × 100, where C represents the corrected absorbance value of the untreated group (infected) and T represents the corrected absorbance value of the compound-treated group. EC_50_ was calculated by the linear regression equation that was obtained by the inhibition rates.

Resazurin blue assay: the bacteria and compounds treatments were the same as for the turbidimeter test. When the bacteria (negative control) grew to OD_595_ ≈ 0.6, about 10 μL resazurin water solution (500 μg/mL) was added to each well in a 96-well plate (200 μL NB, containing bacteria and test compound). After gentle mixing, the mixture was allowed to stand for 10 to 15 min; the color of the uninfected group was blue while the infected group turned red. Similarly, for the compound treatment groups, pink indicated bacterial growth while blue showed an absence of bacterial growth.

### 4.5. Antibacterial Activity Assay in Leaf of Navel Orange

Fully expanded young leaves from healthy Newhall navel orange plants that were planted in a greenhouse were chosen for anti-citrus canker activity assays. Citrus leaves were soaked in 1% sodium hypochlorite for 2 min to disinfect them before rinsing with running water and drying in air. We used a disposable sterilized syringe to prick nine holes in each rectangular square, 2 cm × 2 cm, on the left and right sides of the leaves [31,32]. For the protective assay, we sprayed compound 1, BM, or TC solution (200 μg/mL) evenly on the leaf surface with a spray until drops formed. Then, 24 h after treatment, *Xac* (OD_595_ ≈ 0.2) was inoculated to the above-mentioned wounds, 1 μL per pinhole. The treated leaves were placed in Petri dishes, and the humidity was maintained with absorbent filter paper. For the curative assay, the relevant operations were reversed. All of the samples were cultured at 28 °C in an incubator. Lesions were observed after 7 days of inoculation.

### 4.6. Biofilm Formation

A biofilm formation assay was performed as previously described [33]. Briefly, 10 μL bacterial solution (OD_595_ ≈ 0.6) was added to 100 μL fresh NB medium, and compound 1 was added by the double dilution method. This was then static cultured in an incubator for 36 h. Then, the culture solution was sucked out and gently cleaned with PBS (phosphate-buffered saline, 0.10 mol/L, pH 7.0). Next, we added 100 μL of methanol to each well, fixed it for 15 min, removed the methanol, dried it naturally, added 100 μL of 1.0% crystal violet to each well for dyeing for 5 min, sucked out the excess staining solution, and washed it with running water gently. Finally, we put the 96-well plate upside down on filter paper to dry it and added 200 μL ethanol to dissolve the crystal violet. We then detected the absorbance value at 590 nm.

### 4.7. Apoptosis Detection

An Annexin V-FITC/PI Apoptosis Detection Kit (BD, America) was used, and the experimental operations were carried out following the instructions, as previously reported, with little change [34]. The bacterial NB solution (OD_595_ ≈ 0.6) was centrifuged at 9500 rpm for 5 min and resuspended to OD_595_ ≈ 0.2 with PBS. Then, compound 1 was dissolved in DMSO and added to the bacterial solution, which was incubated at 28 °C and 250 rpm for 12 h. Finally, the solution was incubated with 5 μL of annexin V-FITC for 15 min and with 5 μL of PI for 5 min at room temperature in the dark at 150 rpm. It was then measured by BD FACSCalibur flow cytometry. Data were analyzed using FlowJo V10.

### 4.8. Scanning Electron Microscopy (SEM) Observations

*Xac* cells (OD_595_ ≈ 0.2, NB solution) and compound 1 were co-incubated in NB medium in a shaker (250 rpm) at 28 °C for 12 h. Next, the treated bacteria were centrifuged at 9500 rpm for 5 min at 4 °C and washed with PBS. After that, the *Xac* cells were immobilized using 2.5% glutaraldehyde solution overnight at 4 °C, followed by dehydration with ethanol solution (30%, 50%, 70%, 90%, and 100%). Finally, these samples were freeze-dried, coated with gold, and visualized using a Nova nano SEM 450 instrument [35,36].

### 4.9. Quantitative Proteomics Assay

A tandem mass tags (TMT)-labeled quantitative proteomics assay was carried out according to a method reported elsewhere [37,38,39,40]. For sample preparation, *Xac* cells (OD_595_ ≈ 0.1) and compound 1 were co-incubated in NB medium in a shaker (250 rpm) at 28 °C until the negative control group had grown to OD_595_ ≈ 0.6 (about 10 h). After that, the treated bacteria were collected to yield the total proteins. Then, trypsin digestion, TMT mark, high-performance liquid chromatography, liquid chromatography-mass spectrometry analysis, and protein identification were performed (PTM Biolabs, China, Hangzhou). To further evaluate the response of *Xac* under the stress of compound 1 and to speculate about the potential antibacterial mechanism, an enrichment analysis of the distribution of differential proteins in the gene ontology (GO) secondary annotation, Kyoto Encyclopedia of Genes and Genomes (KEGG), was performed.

### 4.10. RNA Extraction and Real-Time Reverse Transcription Quantitative PCR Assay

For sample preparation, *Xac* cells (OD_595_ ≈ 0.2) and compound 1 were co-incubated in NB medium in a shaker (250 rpm) at 28 °C until all groups had grown to OD_595_ ≈ 0.6. Total RNA of the *Xac* strain was extracted using the Bacteria Total RNA Isolation Kit (Sangon Biotech, China, Shanghai) according to the manufacturer’s protocol. Then, reverse transcription and real-time quantitative RT-PCR were performed with 5 ng of each RNA sample using a One-Step RT-qPCR Kit (Sangon Biotech, China, Shanghai) on a TIAN LONG real-time PCR System. The 16s *rRNA* gene was used as the endogenous reference gene [39]. Relative expressions of each transcript were calculated using the 2^−ΔΔCt^ method [41].

## Figures and Tables

**Table 1:** Inhibition rate (%) of the target compounds against *Xanthomonas axonopodis* pv. *citri* at 100, 50, and 25 μg/mL.

Compound	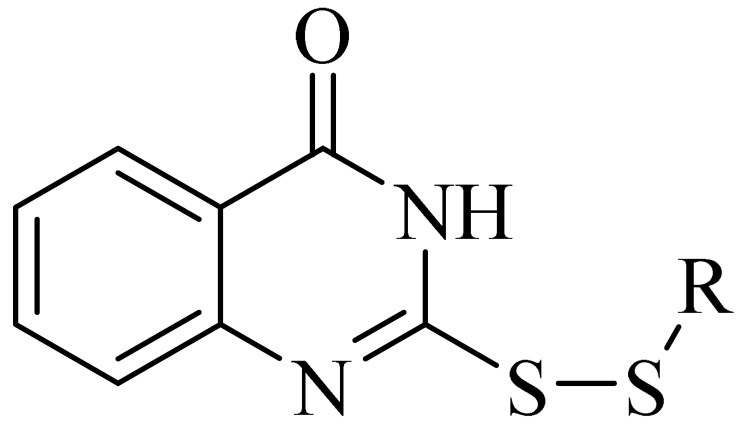	100 μg/mL	50 μg/mL	25 μg/mL
**1**	R: butyl	96.24 ± 3.53	96.11 ± 0.61	98.46 ± 0.30
**2**	R: isobutyl	100.37 ± 1.86	99.31 ± 0.12	98.81 ± 0.20
**3**	R: sec-butyl	99.30 ± 0.08	99.24 ± 0.09	99.37 ± 0.08
**4**	R: tert-butyl	98.67 ± 0.21	98.60 ± 0.44	99.32 ± 0.46
**5**	R: pentyl	97.10 ± 1.08	53.19 ± 3.22	33.55 ± 2.86
**6**	R: isopentyl	69.73 ± 4.65	30.51 ± 2.85	34.55 ± 5.99
**7**	R: hexyl	92.91 ± 0.15	34.53 ± 4.40	38.48 ± 2.30
**8**	R: cyclohexyl	37.26 ± 5.30	33.41 ± 3.69	30.18 ± 6.24
**9**	R: octyl	41.71 ± 3.33	30.48 ± 3.61	23.42 ± 4.01
**10**	R: benzyl	98.75 ± 0.05	98.89 ± 0.19	98.94 ± 0.12
**11**	R: phenyl	98.08 ± 0.65	98.91 ± 0.09	98.74 ± 0.04
**12**	R: p-tolyl	32.14 ± 3.89	39.65 ± 5.91	38.62 ± 2.38
**13**	R: 4-methoxyphenyl	41.45 ± 0.43	31.87 ± 1.65	27.76 ± 2.84
**14**	R: 4-fluorophenyl	87.83 ± 0.16	34.72 ± 0.91	22.13 ± 0.95
**15**	R: 4-chlorophenyl	43.62 ± 2.38	28.44 ± 2.92	28.55 ± 3.20
**16**	R: 4-bromophenyl	38.93 ± 5.38	35.57 ± 2.80	33.93 ± 4.08
**17**	R: 2-fluorophenyl	48.64 ± 2.89	35.51 ± 4.72	26.79 ± 3.35
**18**	R: 2-chlorophenyl	41.05 ± 2.00	32.26 ± 4.63	36.80 ± 1.02
**19**	R: 2-bromophenyl	39.59 ± 3.14	39.19 ± 1.12	39.41 ± 0.67
**20**	R: 3-fluorophenyl	93.97 ± 2.17	88.90 ± 1.86	60.40 ± 1.83
**BM**		67.67 ± 0.42	34.82 ± 2.91	13.96 ± 1.10
**TC**		69.94 ± 0.37	50.86 ± 0.94	16.82 ± 1.79

**Table 2:** The half inhibitory concentration (EC_50_) of the target compounds against *Xanthomonas axonopodis* pv. *citri*.

Compound	Regression Equation	R^2^	EC_50_ (μg/mL)
**1**	y = 4.64x + 3.09	0.966	2.6
**2**	y = 4.78x + 1.48	0.927	5.5
**3**	y = 4.60x + 1.51	0.909	5.7
**4**	y = 4.74x + 0.64	0.944	8.5
**5**	y = 3.85x − 1.03	0.904	36
**10**	y = 4.39x + 2.17	0.903	4.4
**11**	y = 2.19x + 4.07	0.974	2.7
**20**	y = 1.73x + 3.10	0.923	12
**BM**	y = 2.56x + 0.31	0.997	68
**TC**	y = 2.47x + 0.67	0.966	57

**Table 3:** Primers used in this study; the *16s rRNA* gene was used as the endogenous reference gene.

Primer	Sequence
*16s rRNA-F*	CGCTTTCGTGCCTCAGTGTCAGTGTTGG
*16s rRNA-R*	GGCGTAAAGCGTGCGTAGGTGGTGGTT
*dxs-F*	CAAGGGCAAGGGCTACGAACTG
*dxs-R*	TCGGTGTAGGTCGGCTTCTTGG
*dxr-F*	AGTGGTCGATGGGTCCGAAGATC
*dxr-R*	CCGGCAGTCCAAACAGGTGATG
*ispD-F*	TGTTTCGTCGCCATCAACTGATCC
*ispD-R*	CTCCATCGCCATCGCTTCATCG
*ispH-F*	GACGATGCCACGGTGATCTTCAG
*ispH-R*	GCGTCGAACACCTTCAGTCCAC

## Data Availability

Supplementary Material is available online.

## Supplementary Materials

The following supporting information can be downloaded at: https://www.mdpi.com/article/10.3390/ijms231911947/s1.
