# Supplementary material for: Antibacterial Activity of Allicin-Inspired Disulfide Derivatives against Xanthomonas axonopodis pv. citri
Source: Int J Mol Sci. 2022 Oct 8;23(19):11947. doi: 10.3390/ijms231911947 (PMC9569821; doi:10.3390/ijms231911947)
Supplement: Supplementary file 1 [file ijms-23-11947-s001.zip › ijms-1935585-supplementary.pdf]

Supporting Information for

Antibacterial Activity of Allicin-inspired Disulfide Derivatives against *Xanthomonas axonopodis* pv. *citri*

Mei Zhu [1], Yan Li [2], Xuesha Long [2], Congyu Wang [2], Guiping Ouyang [1, 2, *], Zhenchao Wang [2, *]

[1] Center for Research and Development of Fine Chemicals, Guizhou University, Guiyang 550025, China

[2] School of pharmacy, Guizhou University, Guiyang 550025, China

[*] Correspondence: gpouyang@gzu.edu.cn; Tel: +86-851-8830-8270; Fax: +86-851-8830-8270; zcwang@gzu.edu.cn

S1. Synthetic procedures and characterization data of target compounds.

Carbon disulfide (3.03 mL, 50.3 mmol) was added to a stirred solution of potassium hydroxide (1.13 g, 20.1 mmol) in ethanol. The mixture was heated to 30°C for 0.5 h. 2-aminobenzamide (6.85 g, 50.3 mmol) was added to the mixture and heated to 90°C for about 5 h. The reaction was monitored by thin-layer chromatography (TLC). Upon completion, cooled to room temperature and filtered. Then the filter cake was added to the cold water and adjusted the pH to 6 with hydrochloric acid, filtered. The filter cake was washed with water and dried to give 7.18 g 2-thioxo-2,3-dihydroquinazolin-4(1*H*)-one (intermediate 1) with a yield of 80%.

At 0°C under an ice bath, sulfuryl chloride (2.00 mmol) was added dropwise to a solution of the various thiol (1.00 mmol) in dichloromethane, the reaction mixture was stirred for 1 hour at the same temperature, then concentrated under a vacuum to give wine-red liquid various hypochlorothioites (intermediate 2), the crude product can be put into the next reaction without further purification. Take the synthesis procedures of compound 1 as an example, sulfuryl chloride (5.39 mL, 66.5 mmol) was added dropwise to a solution of butane-1-thiol (3.56 mL, 33.3 mmol) in dichloromethane, after reaction in ice bath for about 1 h, the crude products butyl hypochlorothioite are obtained and directly used in the next step.

A mixture of intermediate 1 (2.96 g, 16.6 mmol) in anhydrous ethyl ether, and the newly prepared butyl hypochlorothioite were dissolved with ethyl

ether and slowly drop into the mixture, stirred overnight at room temperature and the reaction was monitored by TLC. After the reaction, the solids were collected by filtration and then purified by column chromatography (petroleum ether and ethyl acetate) to give 2.90 g target compound 1 (2-(butyldisulfanyl) quinazolin-4(3*H*)-one) in 66% yield, the synthetic procedures of other compounds are same as that of compound 1.

*2-(butyldisulfanyl)quinazolin-4(3H)-one (**1**)*: Yield: 66%, mp: 85.9-87.0°C. ^{1}H NMR (400 MHz, $CDCl_3$) δ: 10.00 (s, 1H), 8.27 (dd, J = 8.0, 1.2 Hz, 1H), 7.77 (ddd, J = 8.6, 7.2, 1.5 Hz, 1H), 7.66 – 7.61 (m, 1H), 7.47 (ddd, J = 8.1, 7.1, 1.1 Hz, 1H), 2.87 (t, J = 8.0 Hz, 2H), 1.77 – 1.70 (m, 2H), 1.46 (td, J = 14.9, 7.5 Hz, 2H), 0.95 (t, J = 7.4 Hz, 3H). ^{13}C NMR (101 MHz, $CDCl_3$) δ: 161.23, 152.55, 148.94, 135.28, 126.86, 126.67, 126.58, 120.31, 39.75, 30.74, 21.57, 13.57. ESI-HRMS, m/z $[M + H]^+$ calculated for [$C_{12}H_{15}ON_2S_2$], 267.0620; found, 267.0619.

*2-(isobutyldisulfanyl)quinazolin-4(3H)-one (**2**)*: Yield: 8.5%, mp: 91.5-93.3°C. ^{1}H NMR (400 MHz, DMSO-d_6) δ: 12.64 (s, 1H), 8.08 (ddd, J = 7.8, 4.8, 1.3 Hz, 1H), 7.83 – 7.79 (m, 1H), 7.60 (d, J = 8.2 Hz, 1H), 7.55 – 7.45 (m, 1H), 2.84 (d, J = 6.8 Hz, 1H), 2.54 – 2.47 (m, 2H), 1.10 (dd, J = 12.0, 6.7 Hz, 3H), 1.00 (d, J = 6.7 Hz, 3H).^{13}C NMR (101 MHz, $CDCl_3$) δ: 158.74, 143.53, 133.91, 131.16, 124.83, 65.60, 19.75. ESI-HRMS, m/z $[M + H]^+$ calculated for [$C_{12}H_{15}ON_2S_2$], 267.0620; found, 267.0612.

*2-(sec-butyldisulfanyl)quinazolin-4(3H)-one (**3**)*: Yield: 1.0%, mp: 79.1-81.3°C. ^{1}H NMR (400 MHz, DMSO-d_6) δ: 12.62 (s, 1H), 8.08 (dd, J = 7.9, 0.9 Hz, 1H), 7.88 –

7.78 (m, 1H), 7.59 (d, *J* = 8.2 Hz, 1H), 7.54 – 7.44 (m, 1H), 2.11 – 1.90 (m, 1H), 1.70 – 1.45 (m, 2H), 1.38 – 1.18 (m, 3H), 1.12 – 0.80 (m, 3H). ^{13}C NMR (101 MHz, $CDCl_3$) *δ*: 161.15, 152.89, 148.98, 135.34, 126.94, 126.69, 120.42, 49.76, 28.80, 19.62, 11.57. ESI-HRMS, m/z [M + H]$^+$ calculated for [$C_{12}H_{15}ON_2S_2$], 267.0620; found, 267.0611.

*2-(tert-butyldisulfanyl)quinazolin-4(3H)-one (**4**)*: Yield: 10%, mp: 91.1-93.3°C. ^{1}H NMR (400 MHz, $CDCl_3$) *δ*: 10.76 (s, 1H), 8.26 (t, *J* = 6.6 Hz, 1H), 7.81 – 7.71 (m, 1H), 7.70 – 7.57 (m, 1H), 7.45 – 7.40 (m, 1H), 4.00 – 3.35 (m, 3H), 1.79 – 1.22 (m, 6H). ^{13}C NMR (101 MHz, $CDCl_3$) *δ*: 169.13, 158.82, 156.33, 138.04, 133.06, 130.94, 130.66, 121.12, 54.18, 33.17. ESI-HRMS, m/z [M − H]$^-$ calculated for [$C_{12}H_{13}ON_2S_2$], 265.0464; found, 265.0456.

*2-(pentyldisulfanyl)quinazolin-4(3H)-one (**5**)*: Yield: 1.0%, mp: 67.4-69.6°C. ^{1}H NMR (400 MHz, $CDCl_3$) *δ*: 9.87 (s, 1H), 8.24 (dd, *J* = 8.0, 1.2 Hz, 1H), 7.77 – 7.71 (m, 1H), 7.62 – 7.58 (m, 1H), 7.47 – 7.41 (m, 1H), 5.05 (dd, *J* = 7.7, 5.9 Hz, 1H), 2.83 (t, *J* = 5.8 Hz, 1H), 2.18 – 2.06 (m, 2H), 1.71 (q, *J* = 7.5 Hz, 1H), 1.41 – 1.28 (m, 3H), 0.93 – 0.88 (m, 3H). ^{13}C NMR (101 MHz, $CDCl_3$) *δ*: 161.23, 152.55, 148.94, 135.28, 126.86, 126.67, 126.58, 120.31, 39.75, 30.74, 21.57, 13.57. ESI-HRMS, m/z [M + H]$^+$ calculated for [$C_{13}H_{17}ON_2S_2$], 281.0777; found, 281.0777.

*2-(isopentyldisulfanyl)quinazolin-4(3H)-one (**6**)*: Yield: 75%, mp: 109.7-112.1°C. ^{1}H NMR (400 MHz, $CDCl_3$) *δ*: 8.28 (d, *J* = 7.8 Hz, 1H), 8.00 (d, *J* = 8.0 Hz, 1H), 7.87 (t, *J* = 7.4 Hz, 1H), 7.59 (t, *J* = 7.5 Hz, 1H), 3.73 (q, *J* = 7.0 Hz, 2H), 1.25 (t, *J* = 7.0 Hz, 3H), 1.03 – 0.90 (m, 6H). ^{13}C NMR (101 MHz, $CDCl_3$) *δ*: 136.61, 128.63, 127.58,

123.26, 119.31, 69.59, 46.90, 26.28, 22.43, 21.36. ESI-HRMS, m/z $[M + H]^+$ calculated for $[C_{13}H_{17}ON_2S_2]$, 281.0777; found, 281.0775.

2-(hexyldisulfanyl)quinazolin-4(3H)-one (**7**): Yield: 18%, mp: 76.4-79.3°C. ^1H NMR (400 MHz, $CDCl_3$) *δ*: 9.84 (s, 1H), 8.26 (dd, *J* = 8.0, 1.2 Hz, 1H), 7.78 – 7.74 (m, 1H), 7.65 – 7.61 (m, 1H), 7.49 – 17.45 (m, 1H), 5.06 (dd, *J* = 7.5, 6.0 Hz, 1H), 3.73 (q, *J* = 7.0 Hz, 1H), 2.15 – 12.12 (m, 2H), 1.35 – 1.30 (m, 3H), 1.25 (t, *J* = 7.0 Hz, 3H), 0.93 – 0.86 (m, 3H). ^{13}C NMR (101 MHz, DMSO-d_6) *δ*: 161.78, 153.77, 148.45, 135.33, 127.20, 126.84, 126.61, 120.98, 70.95, 38.21, 30.79, 26.19, 22.35, 14.23. ESI-HRMS, m/z $[M - H]^-$ calculated for $[C_{14}H_{17}ON_2S_2]$, 293.0777; found, 293.0775.

2-(cyclohexyldisulfanyl)quinazolin-4(3H)-one (**8**): Yield: 2.8%, mp: 128.7-131.1°C. ^1H NMR (400 MHz, DMSO-d_6) *δ*: 12.53 (s, 1H), 8.07 (dd, *J* = 7.9, 1.3 Hz, 1H), 7.83 – 7.78 (m, 1H), 7.60 (d, *J* = 7.7 Hz, 1H), 7.52 – 7.45 (m, 1H), 3.13 – 3.04 (m, 1H), 2.05 – 1.93 (m, 2H), 1.77 – 1.67 (m, 2H), 1.45 – 1.15 (m, 6H). ^{13}C NMR (101 MHz, DMSO-d_6) *δ*: 148.69, 135.36, 126.93, 126.90, 126.60, 120.83, 49.68, 32.37, 25.69, 25.45. ESI-HRMS, m/z $[M + H]^+$ calculated for $[C_{14}H_{17}ON_2S_2]$, 293.0777; found, 293.0776.

2-(octyldisulfanyl)quinazolin-4(3H)-one (**9**): Yield: 56%, mp: 104.5-106.7°C. ^1H NMR (400 MHz, $CDCl_3$) *δ*: 8.27 (d, *J* = 7.8 Hz, 1H), 8.13 – 7.80 (m, 2H), 7.64 – 7.50 (m, 1H), 3.00 – 2.86 (m, 1H), 2.22 – 2.10 (m, 1H), 1.81 – 1.50 (m, 2H), 1.46 – 1.17 (m, 10H), 0.88 (q, *J* = 6.7 Hz, 3H). ^{13}C NMR (101 MHz, $CDCl_3$) *δ*: 135.41, 135.35, 127.15, 127.00, 126.95, 126.85, 126.72, 120.32, 67.99, 57.12, 55.89, 48.47, 31.61,

31.54, 24.92, 21.09. ESI-HRMS, m/z [M − H]$^-$ calculated for [$C_{16}H_{21}ON_2S_2$], 321.1090; found, 321.1079.

*2-(benzyldisulfanyl)quinazolin-4(3H)-one (**10**)*: Yield: 9.4%, mp: 131.6-134.1°C. ^{1}H NMR (400 MHz, DMSO-*d*$_6$) *δ*: 12.46 (s, 1H), 8.06 (dd, *J* = 7.9, 1.4 Hz, 1H), 7.83 – 7.79 (m, 1H), 7.64 (d, *J* = 7.8 Hz, 1H), 7.54 – 7.45 (m, 1H), 7.40 – 7.36 (m, 2H), 7.33 – 7.26 (m, 2H), 7.23 – 7.18 (m, 1H), 4.22 (s, 2H). ^{13}C NMR (101 MHz, DMSO-*d*$_6$) *δ*: 136.74, 135.26, 130.04, 128.86, 128.00, 126.86, 126.56, 120.80, 42.42. ESI-HRMS, m/z [M + Na]$^+$ calculated for [$C_{15}H_{12}ON_2S_2Na$], 323.0283; found, 323.0283.

*2-(phenyldisulfanyl)quinazolin-4(3H)-one (**11**):* Yield: 84%, mp: 138.2-140.4°C. ^{1}H NMR (400 MHz, $CDCl_3$) *δ*: 9.89 (s, 1H), 8.26 (dd, *J* = 8.0, 1.3 Hz, 1H), 7.79 – 7.75 (m, 1H), 7.65 (d, *J* = 7.8 Hz, 1H), 7.59 – 7.55 (m, 2H), 7.51 – 7.44 (m, 1H), 7.42 – 7.33 (m, 3H). ^{13}C NMR (101 MHz, $CDCl_3$) *δ*: 161.13, 151.64, 148.80, 135.31, 133.62, 129.80, 128.98, 128.67, 126.89, 126.87, 126.70, 120.44. ESI-HRMS, m/z [M + H]$^+$ calculated for [$C_{14}H_{11}ON_2S_2$], 287.0307; found, 287.0300.

*2-(p-tolyldisulfanyl)quinazolin-4(3H)-one (**12**)*: Yield: 3.8%, mp: 160.3-161.8°C. ^{1}H NMR (400 MHz, DMSO-*d*$_6$) *δ*: 12.81 (s, 1H), 8.07 (dd, *J* = 7.9, 1.3 Hz, 1H), 7.85 – 7.77 (m, 1H), 7.61 (dd, *J* = 20.1, 8.1 Hz, 3H), 7.53 – 7.46 (m, 1H), 7.21 (d, *J* = 8.1 Hz, 2H), 2.28 (s, 3H). ^{13}C NMR (101 MHz, DMSO-*d*$_6$) *δ*: 139.20, 135.35, 132.30, 131.11, 130.50, 127.03, 126.63, 21.15. ESI-HRMS, m/z [M + Na]$^+$ calculated for [$C_{15}H_{12}ON_2S_2Na$], 323.0283; found, 323.0285.

*2-((4-methoxyphenyl)disulfanyl)quinazolin-4(3H)-one (**13**)*: Yield: 59%, mp: 158.7-161.1°C. ^{1}H NMR (400 MHz, $CDCl_3$) *δ*: 9.99 (s, 1H), 8.24 (dd, *J* = 8.0, 1.3 Hz, 1H),

7.76 – 7.70 (m, 1H), 7.59 (d, *J* = 8.1 Hz, 1H), 7.52 – 7.49 (m, 2H), 7.46 – 7.41 (m, 1H), 6.87 – 6.84 (m, 2H), 3.78 (s, 3H). ^{13}C NMR (101 MHz, $CDCl_3$) δ: 161.25, 161.05, 152.24, 148.86, 135.30, 132.98, 126.87, 126.79, 126.66, 124.46, 120.35, 115.42, 55.52. ESI-HRMS, m/z [M + H]$^+$ calculated for [$C_{15}H_{13}O_2N_2S_2$], 317.0413; found, 317.0411.

*2-((4-fluorophenyl)disulfanyl)quinazolin-4(3H)-one (**14**)*: Yield: 40%, mp: 128.7-131.3°C. ^{1}H NMR (400 MHz, $CDCl_3$) δ: 8.25 (s, 1H), 7.79 (d, *J* = 7.2 Hz, 1H), 7.61 – 7.36 (m, 4H), 7.13 – 6.96 (m, 2H). ^{13}C NMR (101 MHz, DMSO-d_6) δ: 190.76, 162.52, 160.59, 159.93, 144.39, 132.21, 131.60, 130.40, 123.74, 123.55, 115.90, 112.46, 112.27. ESI-HRMS, m/z [M − H]$^-$ calculated for [$C_{14}H_8ON_2S_2F$], 303.0057; found, 303.0073.

*2-((4-chlorophenyl)disulfanyl)quinazolin-4(3H)-one (**15**)*: Yield: 75%, mp: 177.8-179.5°C. ^{1}H NMR (400 MHz, $CDCl_3$) δ: 8.27 (dd, *J* = 8.0, 1.3 Hz, 1H), 7.81 (td, *J* = 7.8, 7.2, 1.5 Hz, 1H), 7.69 (d, *J* = 7.6 Hz, 2H), 7.57 – 7.48 (m, 3H), 7.35 (d, *J* = 8.6 Hz, 1H). ^{13}C NMR (101 MHz, $CDCl_3$) δ: 161.14, 151.13, 148.70, 135.42, 133.47, 132.51, 130.42, 129.86, 129.38, 128.23, 127.07, 127.00, 126.73, 120.53. ESI-HRMS, m/z [M − H]$^-$ calculated for [$C_{14}H_8ON_2S_2Cl$], 318.9761; found, 318.9775.

*2-((4-bromophenyl)disulfanyl)quinazolin-4(3H)-one (**16**)*: Yield: 53%, mp: 194.7-195.5°C. ^{1}H NMR (400 MHz, $CDCl_3$) δ: 9.77 (s, 1H), 8.24 (dd, *J* = 8.0, 1.2 Hz, 1H), 7.80 – 7.75 (m, 1H), 7.63 (d, *J* = 8.2 Hz, 1H), 7.51 – 7.41 (m, 5H). ^{13}C NMR (101 MHz, $CDCl_3$) δ: 161.16, 151.15, 148.74, 135.48, 132.97, 130.29, 127.13, 126.99,

126.80, 123.43, 120.49. ESI-HRMS, m/z [M − H]$^-$ calculated for [$C_{14}H_8ON_2S_2Br$], 362.9256; found, 362.9273.

2-((2-fluorophenyl)disulfanyl)quinazolin-4(3H)-one (***17***): Yield: 85%, mp: 152.3-153.6°C. ^{1}H NMR (400 MHz, $CDCl_3$) *δ*: 10.08 (s, 1H), 8.26 (dd, *J* = 7.9, 1.1 Hz, 1H), 7.80 – 7.70 (m, 1H), 7.68 – 7.59 (m, 2H), 7.49 – 7.36 (m, 2H), 7.20 – 7.11 (m, 2H). ^{13}C NMR (101 MHz, $CDCl_3$) *δ*: 162.48, 161.21, 160.50, 151.83, 148.69, 135.36, 133.51, 132.32, 127.00, 126.65, 125.53, 120.47, 116.80, 116.63. ESI-HRMS, m/z [M + H]$^+$ calculated for [$C_{14}H_{10}ON_2S_2$ F], 305.0213; found, 305.0208.

2-((2-chlorophenyl)disulfanyl)quinazolin-4(3H)-one (***18***): Yield: 67%, mp: 157.6-159.4°C. ^{1}H NMR (400 MHz, $CDCl_3$) *δ*: 9.85 (s, 1H), 8.22 (dd, *J* = 7.9, 1.4 Hz, 1H), 7.75 (td, *J* = 7.8, 7.3, 1.5 Hz, 1H), 7.65 – 7.60 (m, 2H), 7.47 – 7.40 (m, 2H), 7.30 – 7.25 (m, 2H). ^{13}C NMR (101 MHz, $CDCl_3$) *δ*: 161.16, 151.15, 148.70, 135.44, 133.46, 132.50, 130.42, 129.86, 129.36, 128.23, 127.08, 127.00, 126.73, 120.53. ESI-HRMS, m/z [M + H]$^+$ calculated for [$C_{14}H_{10}ON_2S_2Cl$], 320.9918; found, 320.9908.

2-((2-bromophenyl)disulfanyl)quinazolin-4(3H)-one (***19***)*:* Yield: 83%, mp: 165.8-167.1°C. ^{1}H NMR (400 MHz, $CDCl_3$) *δ*: 8.23 (d, *J* = 7.8 Hz, 1H), 7.78 (t, *J* = 7.3 Hz, 1H), 7.70 – 7.63 (m, 2H), 7.63 – 7.57 (m, 1H), 7.47 (t, *J* = 7.5 Hz, 1H), 7.36 (t, *J* = 7.4 Hz, 1H), 7.20 (t, *J* = 7.5 Hz, 1H). ^{13}C NMR (101 MHz, $CDCl_3$) *δ*: 161.18, 151.12, 148.66, 135.44, 134.46, 133.58, 129.77, 128.83, 128.76, 127.10, 127.01, 126.72, 122.59, 120.53. ESI-HRMS, m/z [M − H]$^-$ calculated for [$C_{14}H_8ON_2S_2$ Br], 362.9256; found, 362.9272.

2-((3-fluorophenyl)disulfanyl)quinazolin-4(3H)-one (**20**): Yield: 78%, mp: 140.7-143.3°C. ^1H NMR (400 MHz, $CDCl_3$) δ: 9.83 (s, 1H), 8.26 – 8.19 (m, 1H), 7.79 – 7.73 (m, 1H), 7.63 (d, *J* = 8.1 Hz, 1H), 7.45 (t, *J* = 7.4 Hz, 1H), 7.35 – 7.25 (m, 3H), 7.04 – 6.97 (m, 1H). ^{13}C NMR (101 MHz, $CDCl_3$) δ: 161.20, 151.08, 135.52, 131.29, 131.23, 127.16, 127.00, 126.78, 123.69, 120.49, 116.17, 116.00, 115.30, 115.10. ESI-HRMS, m/z $[M + H]^+$ calculated for $[C_{14}H_{10}ON_2S_2F]$, 305.0213; found, 305.0203.

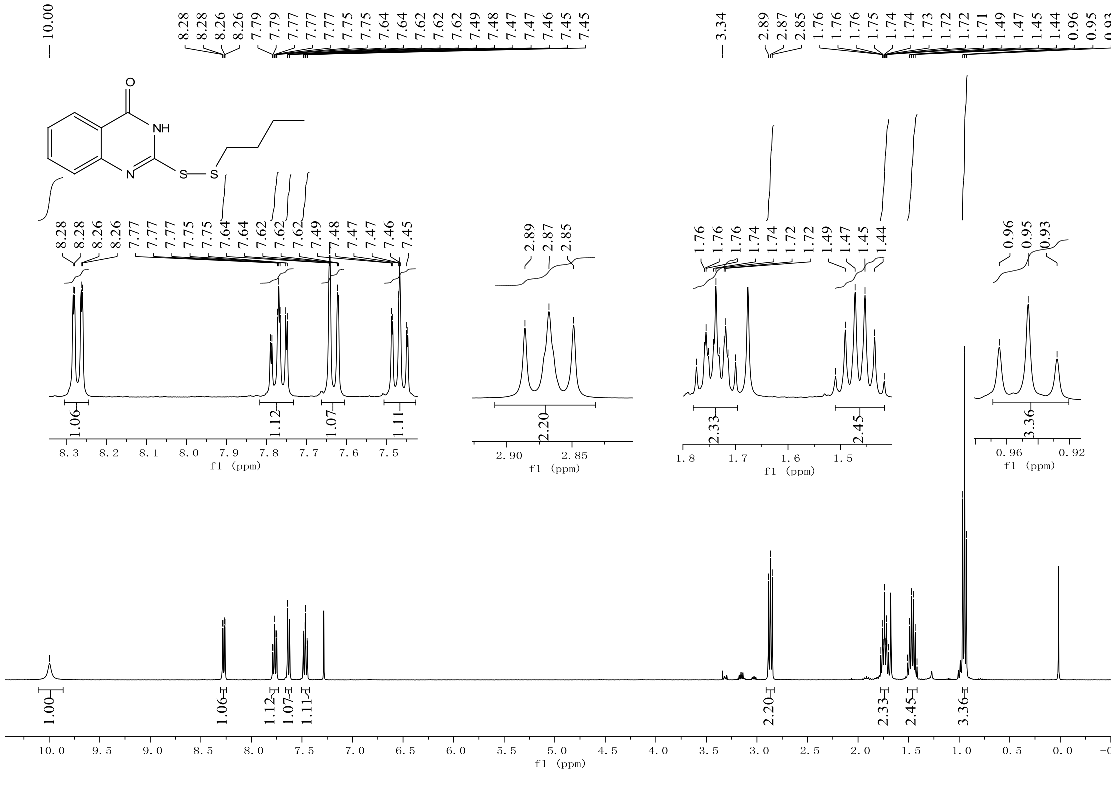

^1H NMR of compound 1

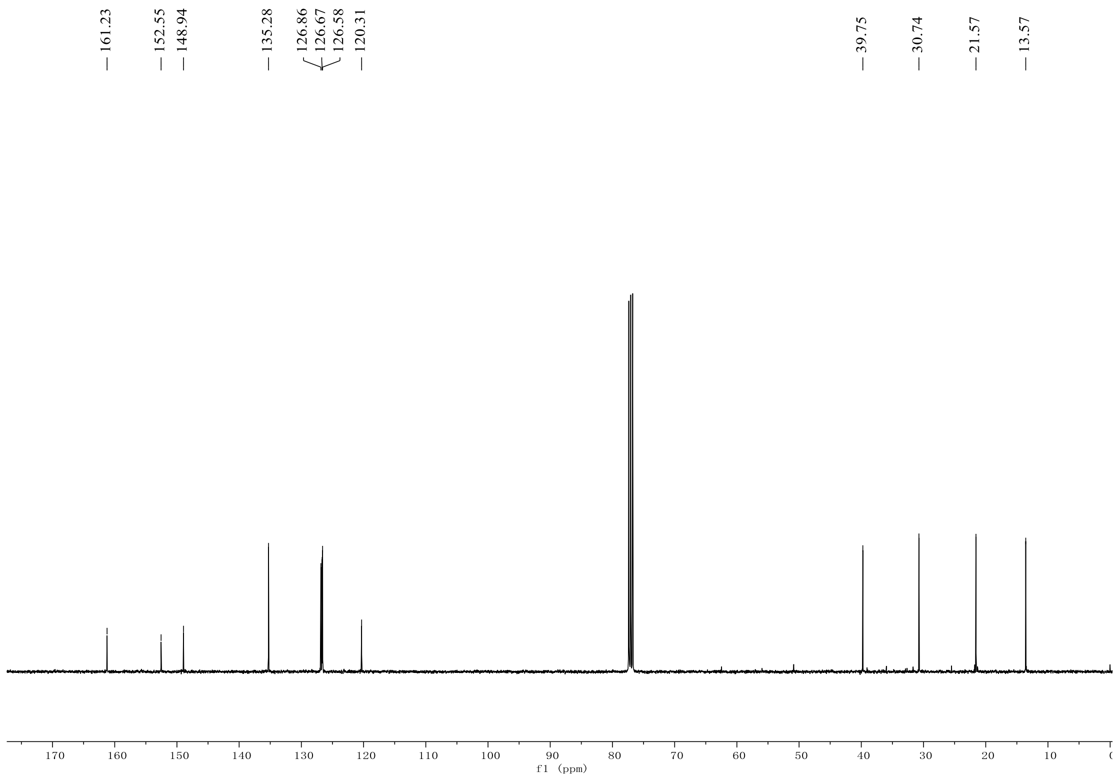

^{13}C NMR of compound 1

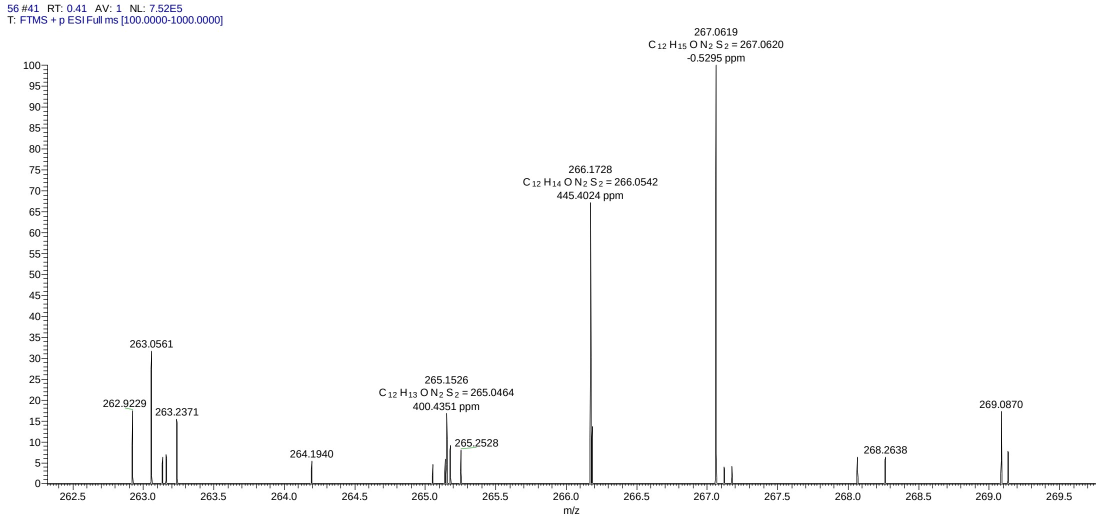

HRMS of compound 1 [M+1]

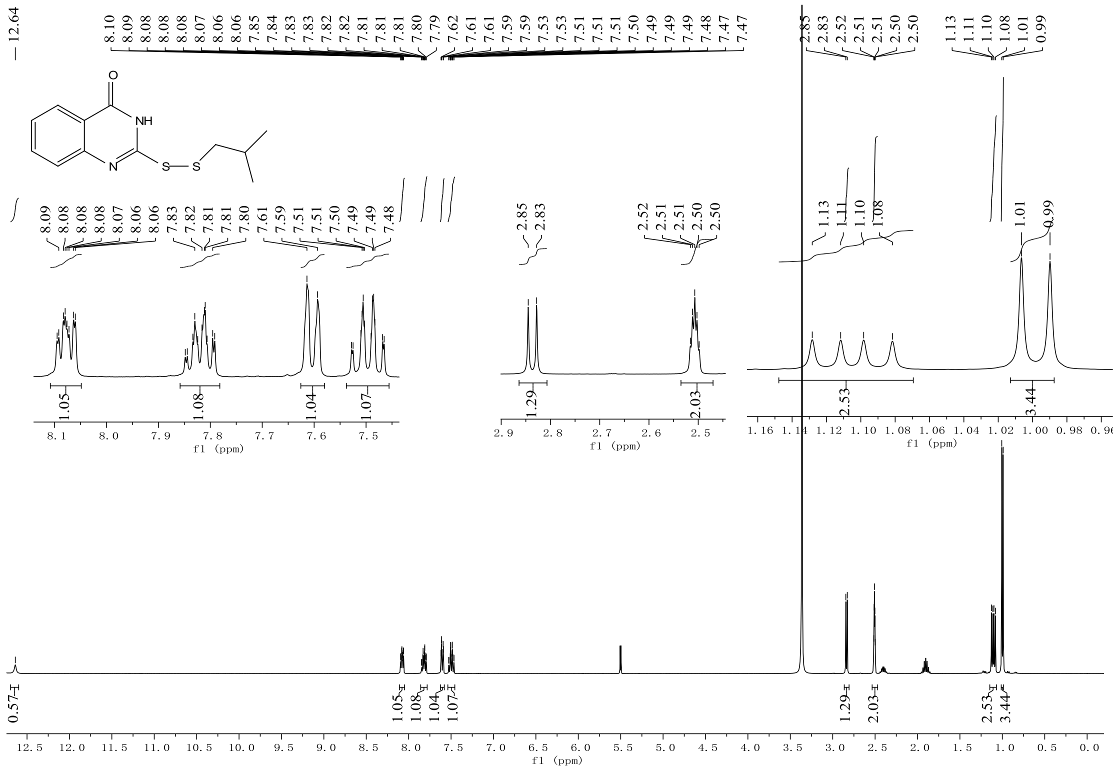

¹H NMR of compound 2

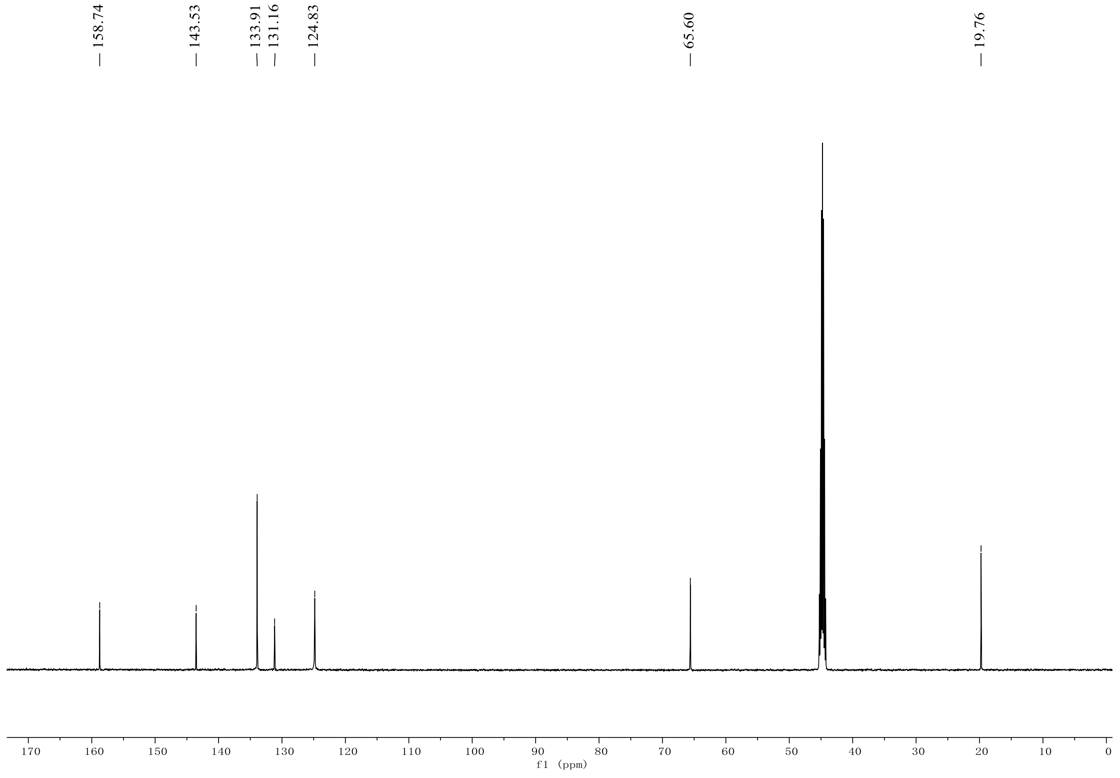

¹³C NMR of compound 2

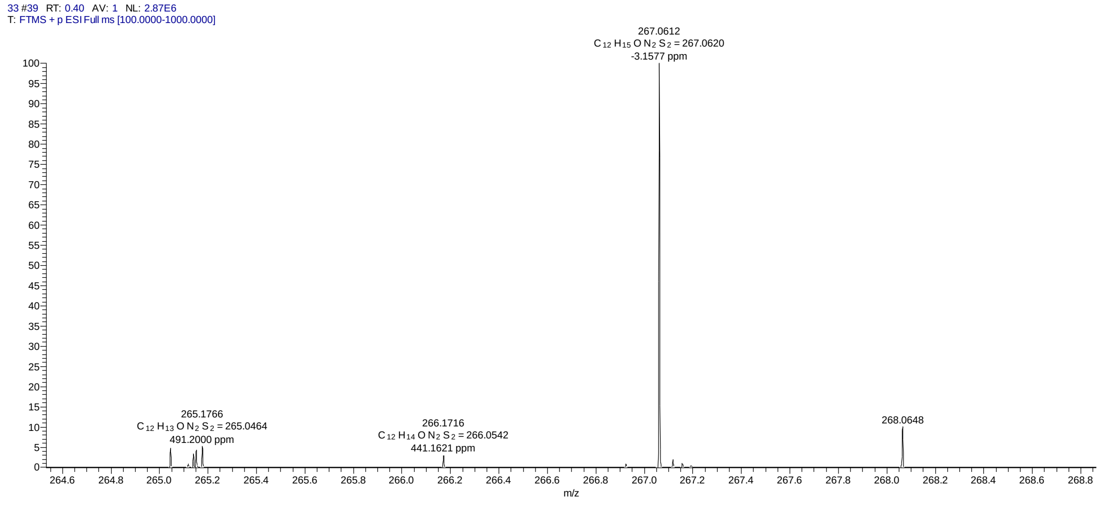

HRMS of compound 2 [M+1]

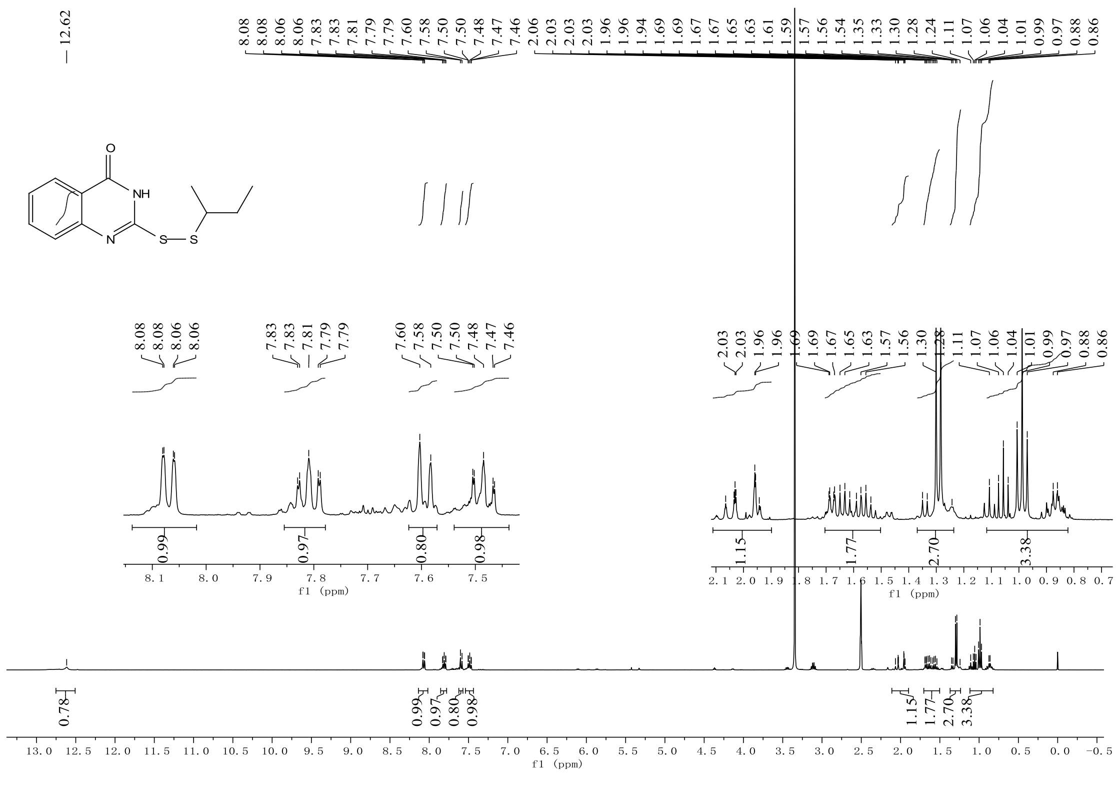

^{1}H NMR of compound 3

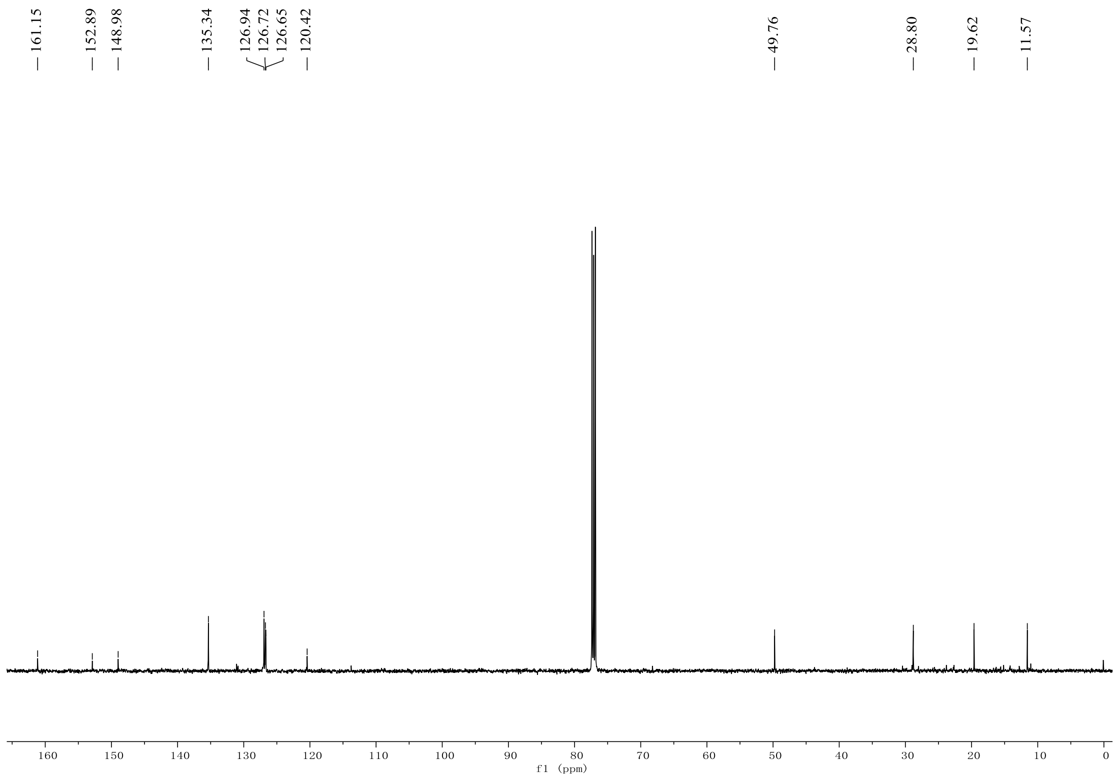

^{13}C NMR of compound 3

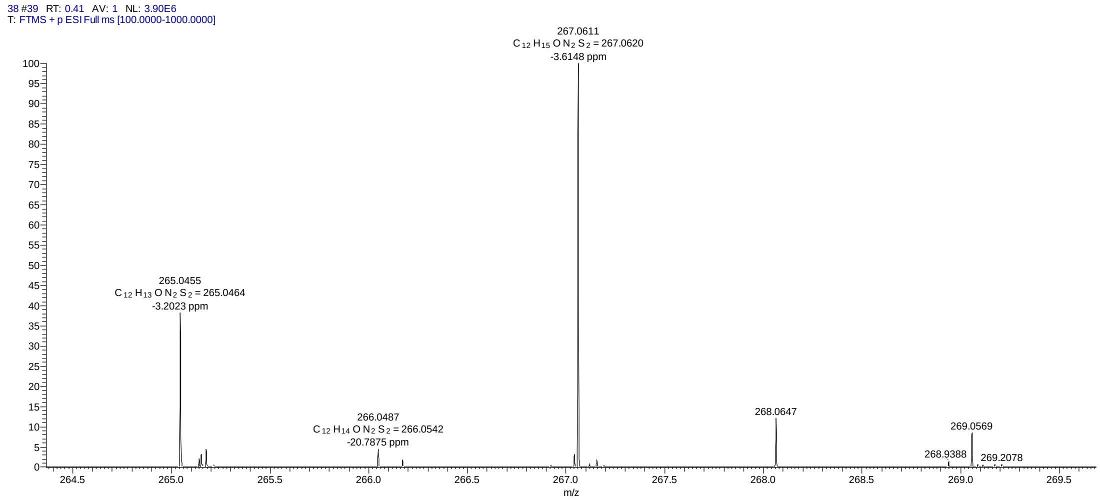

HRMS of compound 3 [M+1]

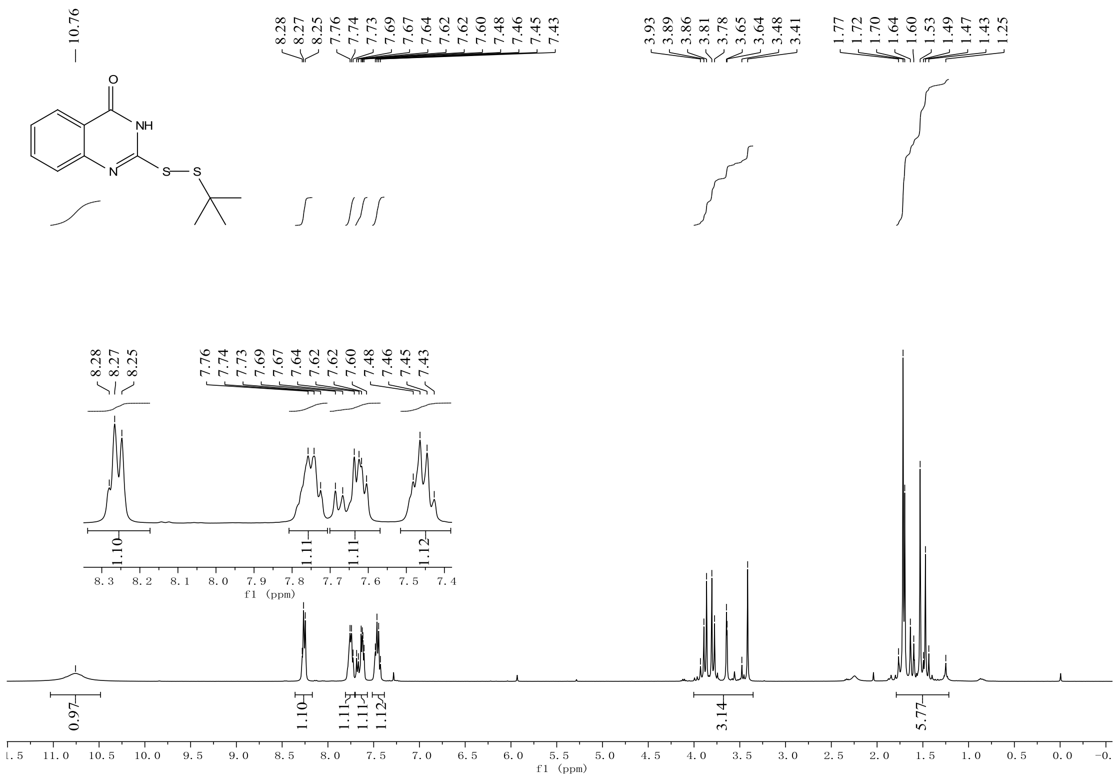

¹H NMR of compound 4

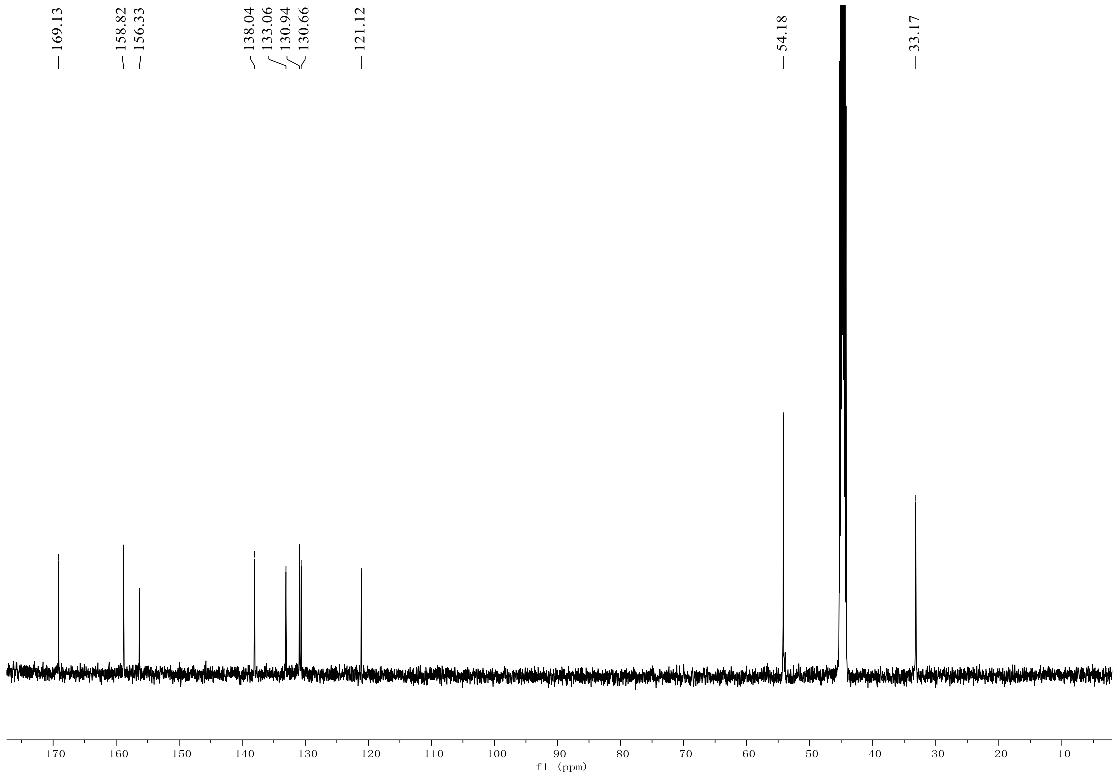

¹³C NMR of compound 4

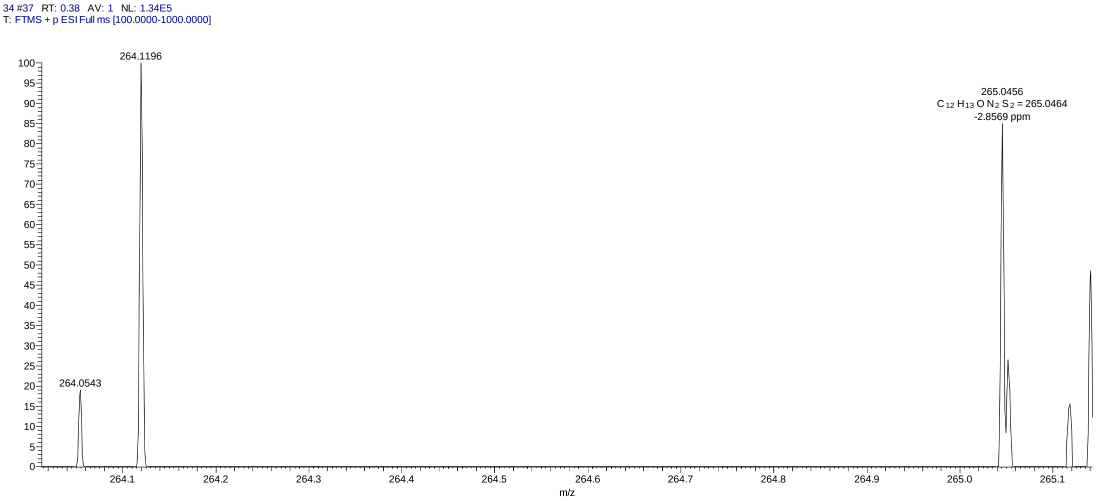

HRMS of compound 4 [M−1]

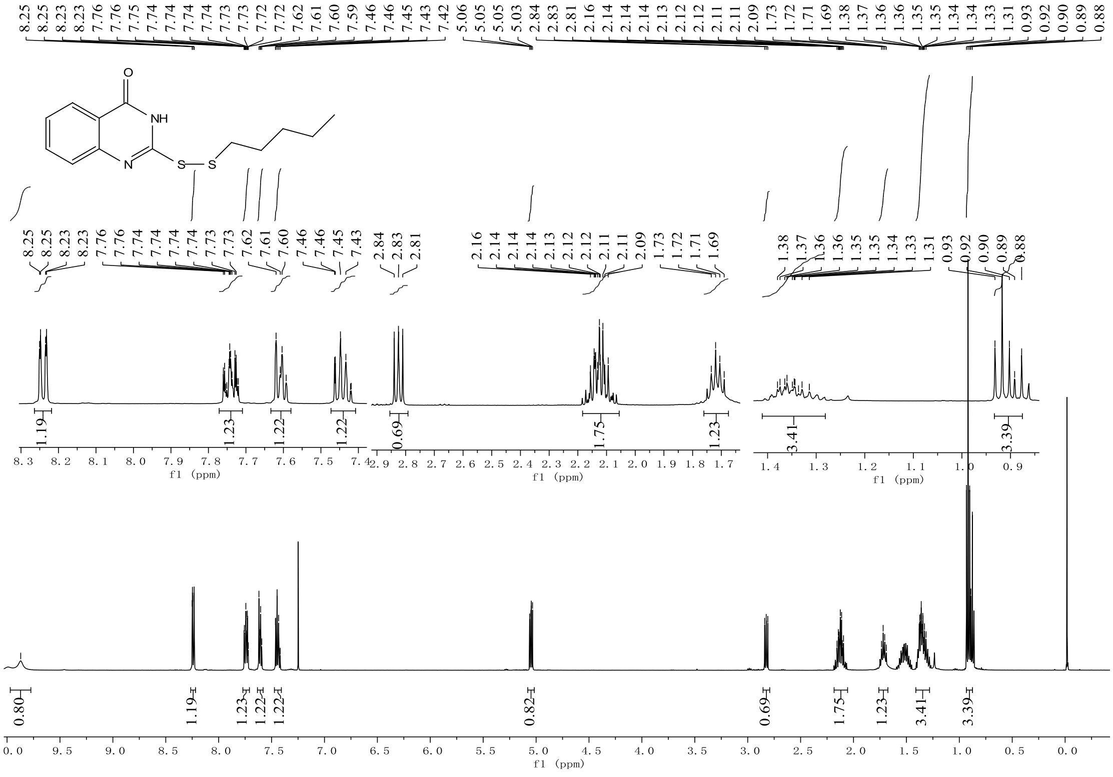

^{1}H NMR of compound 5

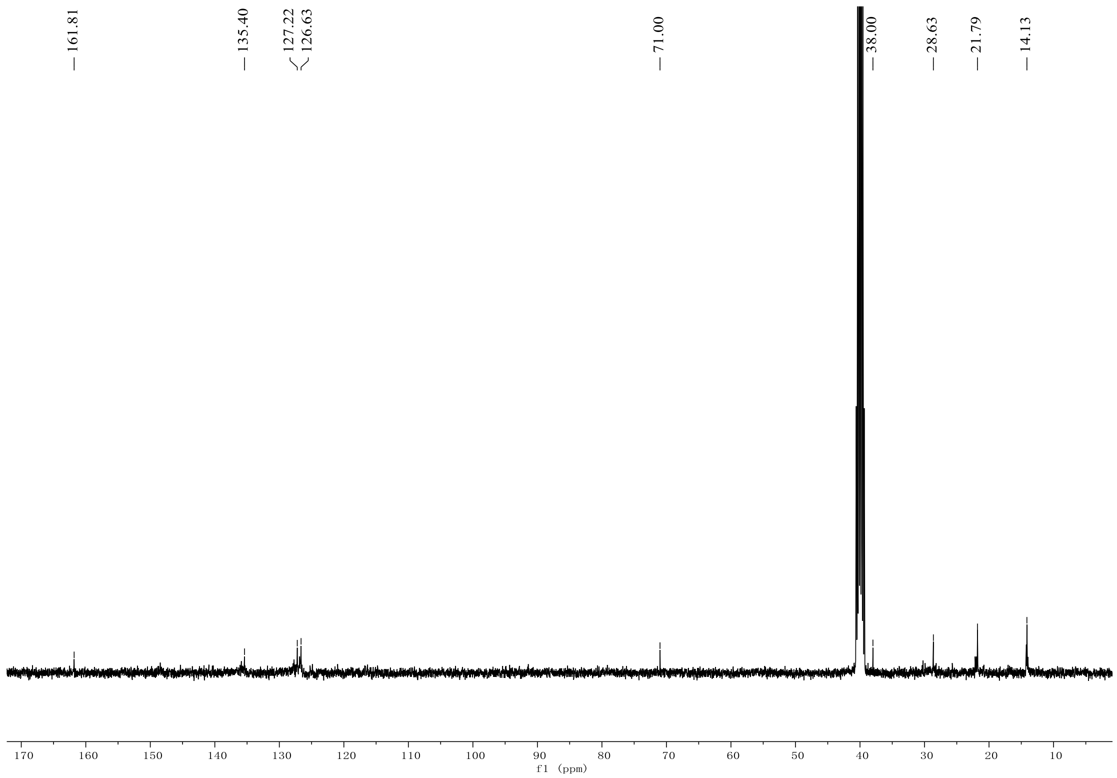

^{13}C NMR of compound 5

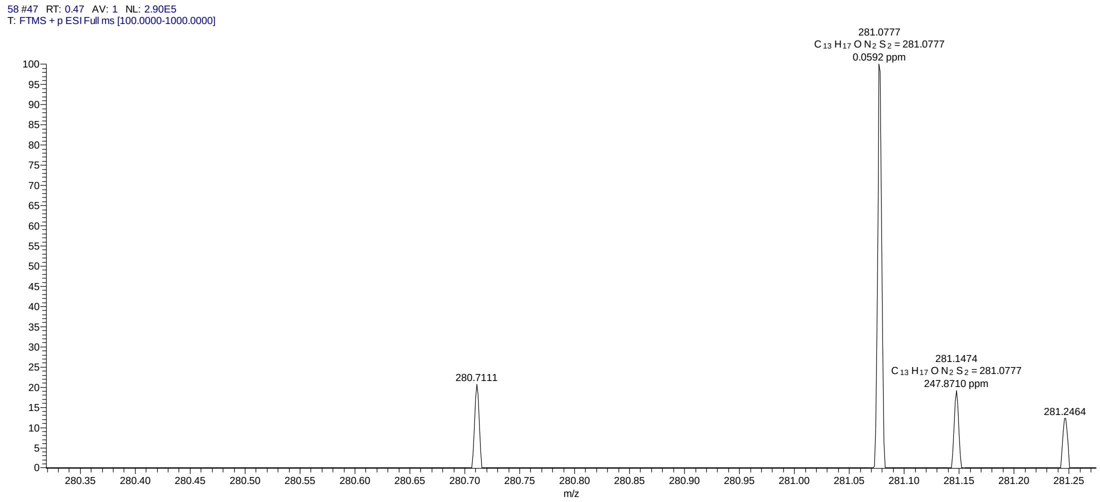

HRMS of compound 5 [M+1]

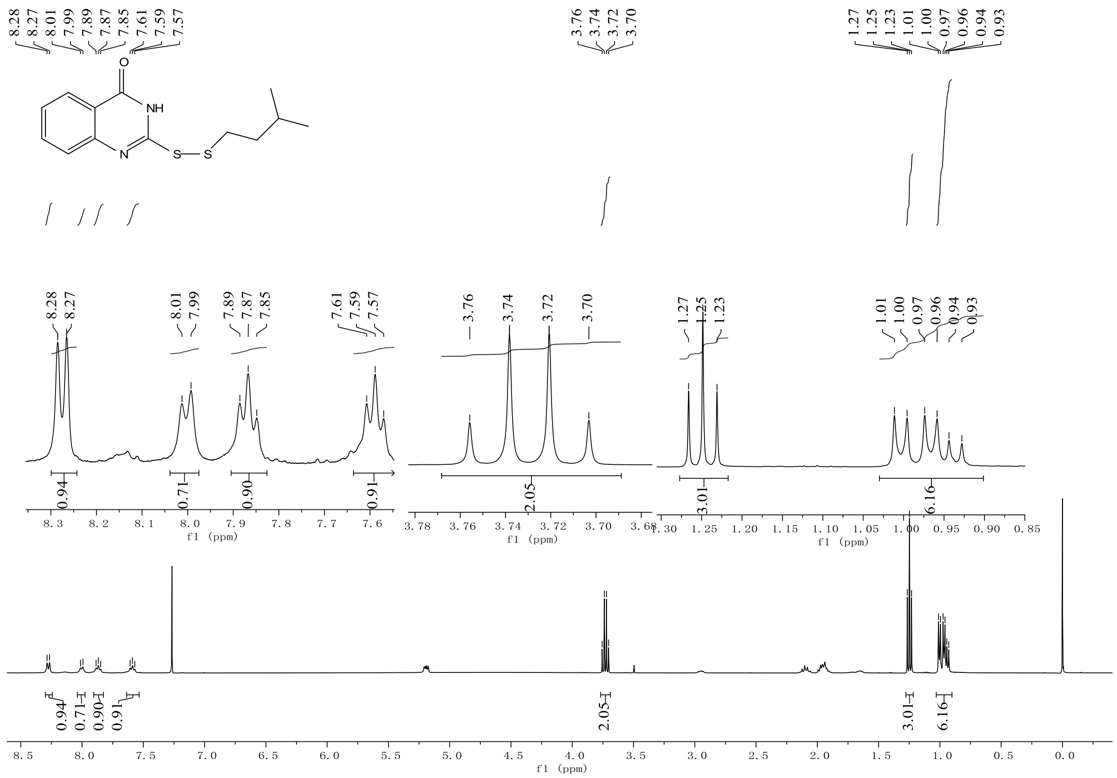

^{1}H NMR of compound 6

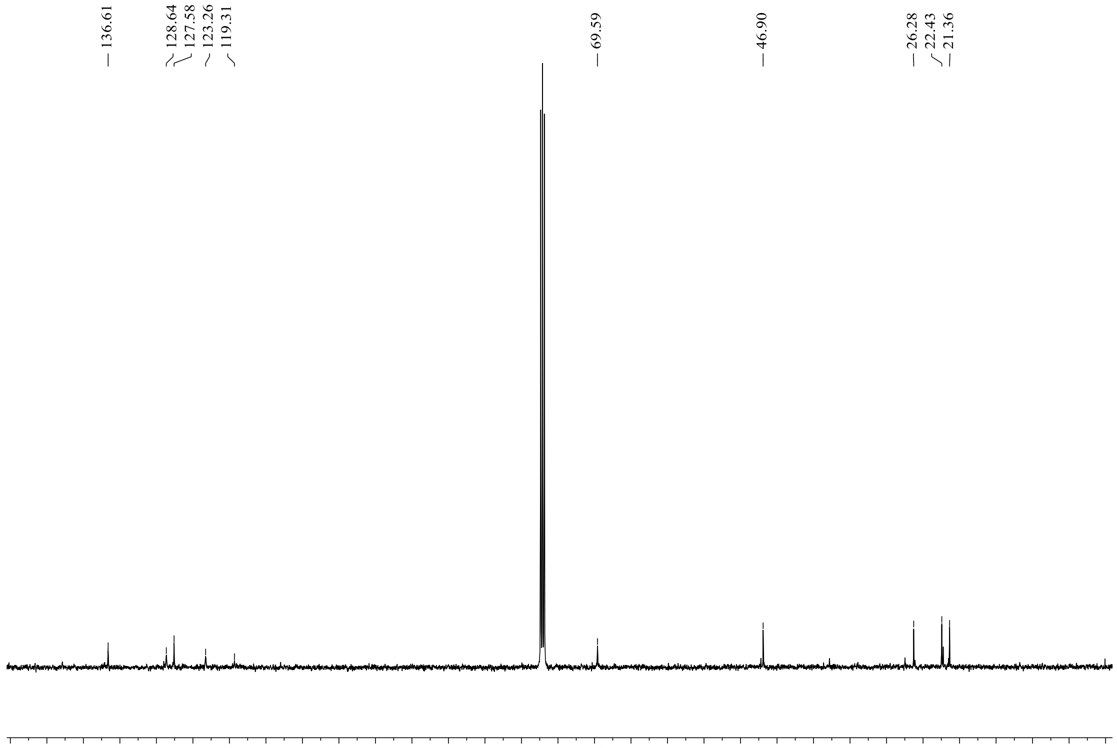

^{13}C NMR of compound 6

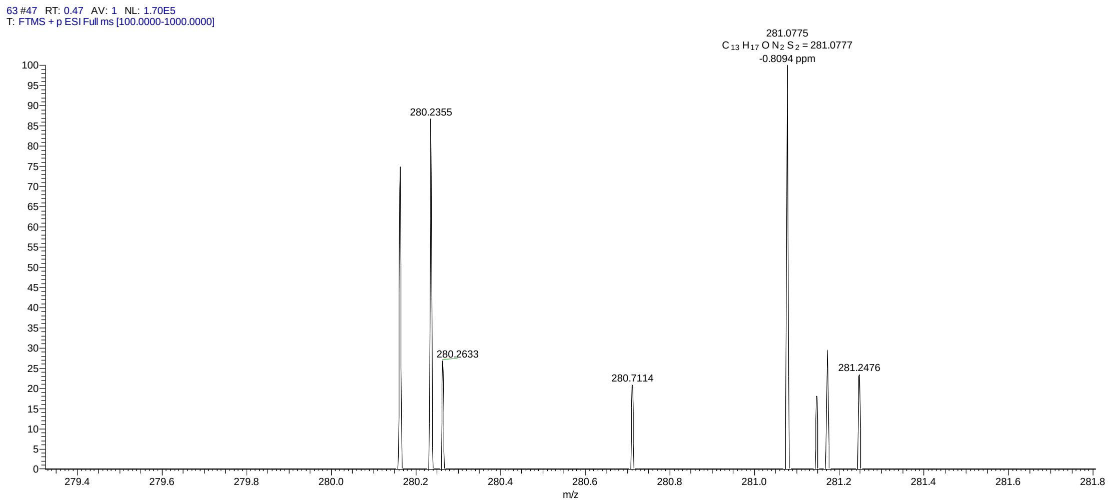

HRMS of compound 6 [M+1]

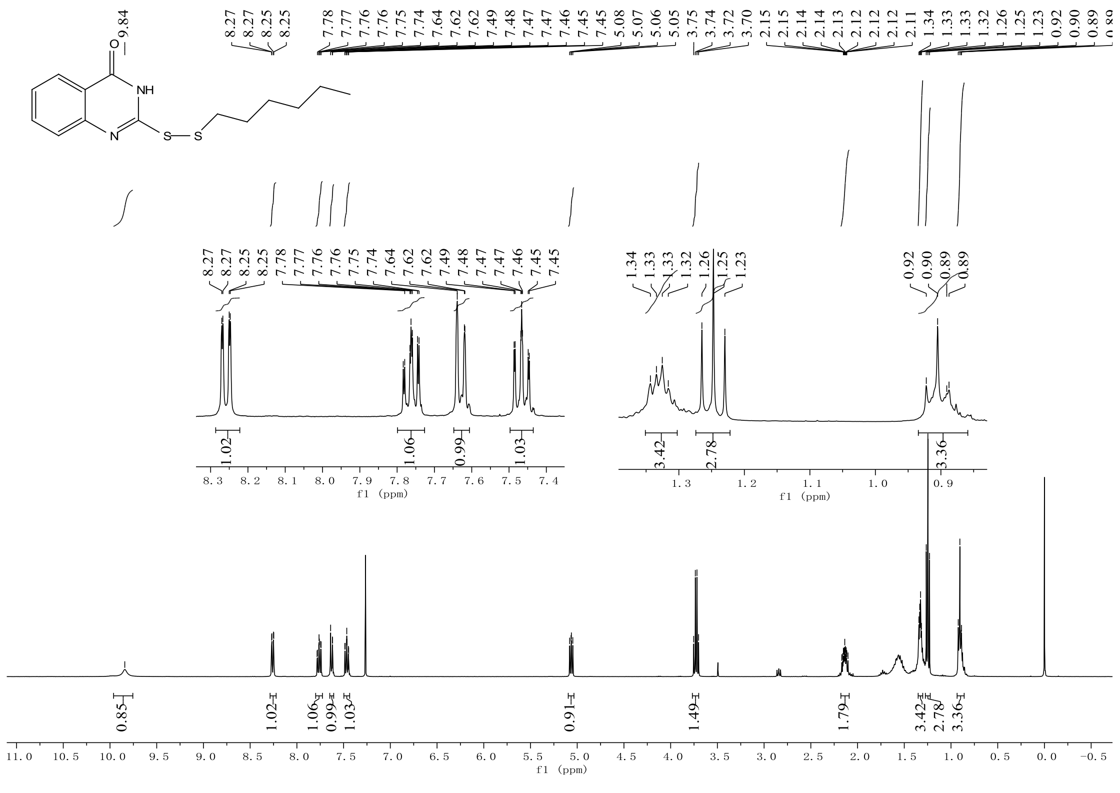

^{1}H NMR of compound 7

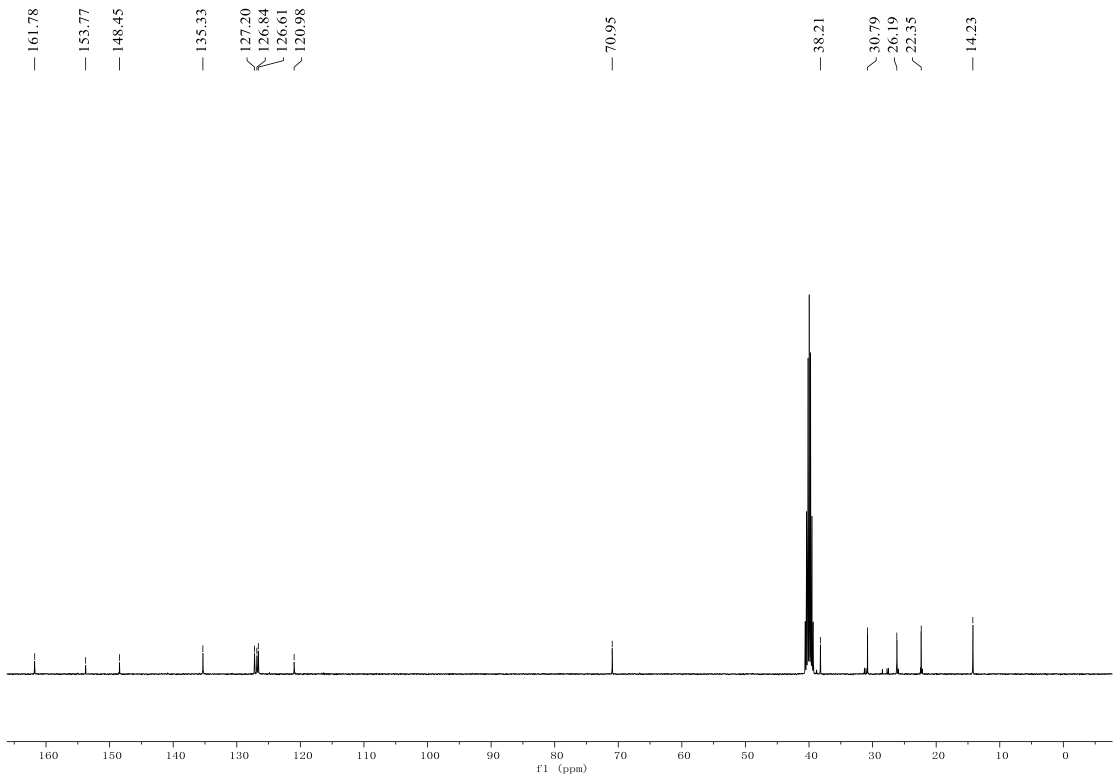

^{13}C NMR of compound 7

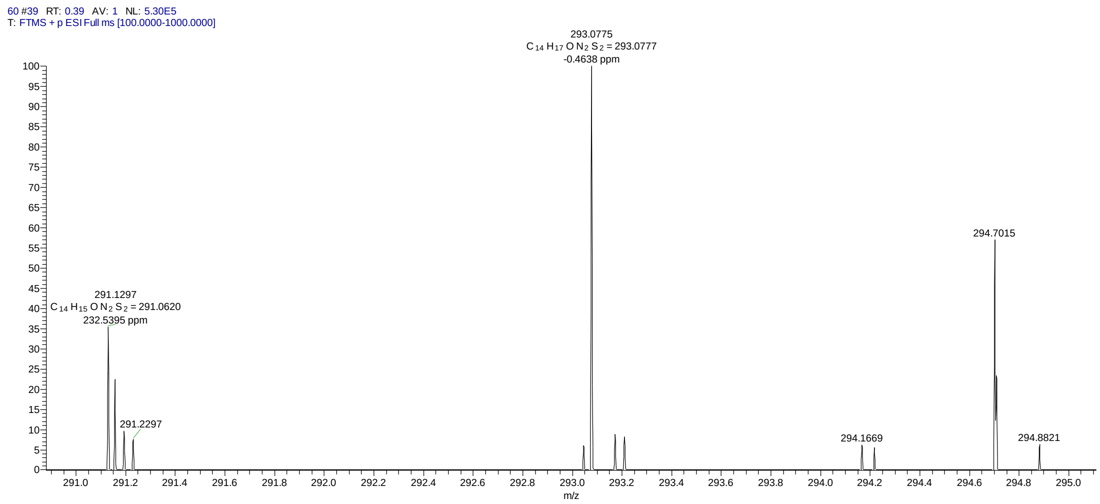

HRMS of compound 7 [M−1]

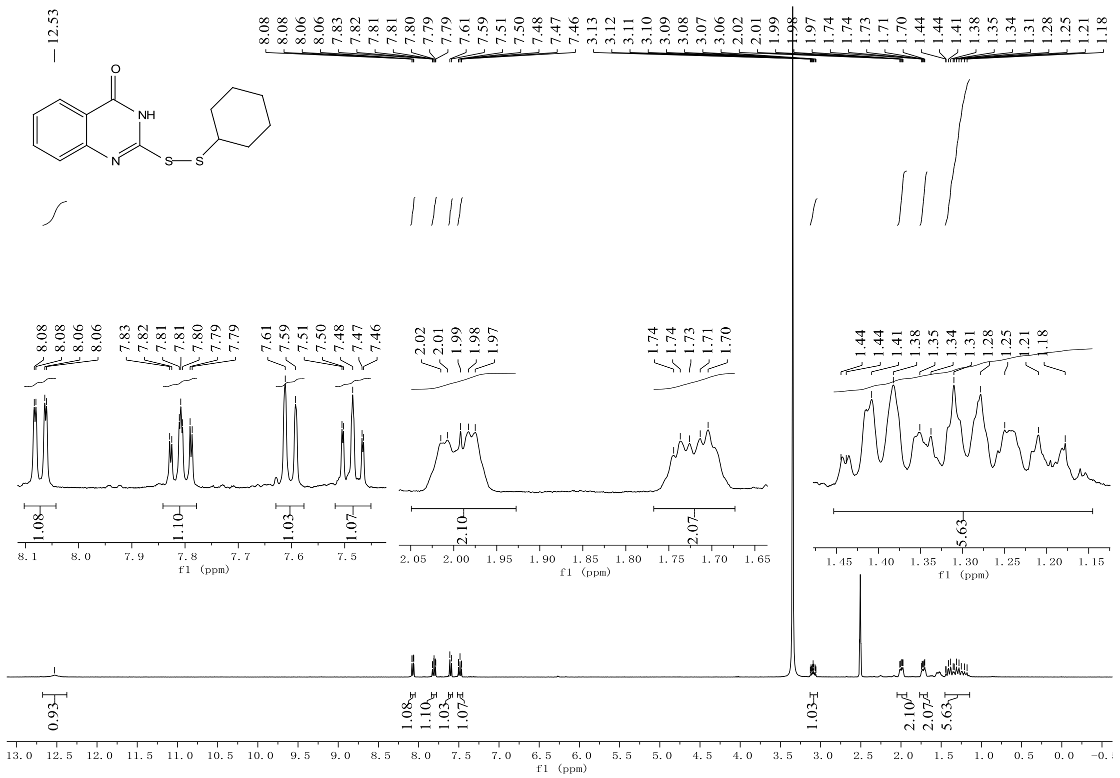

^{1}H NMR of compound 8

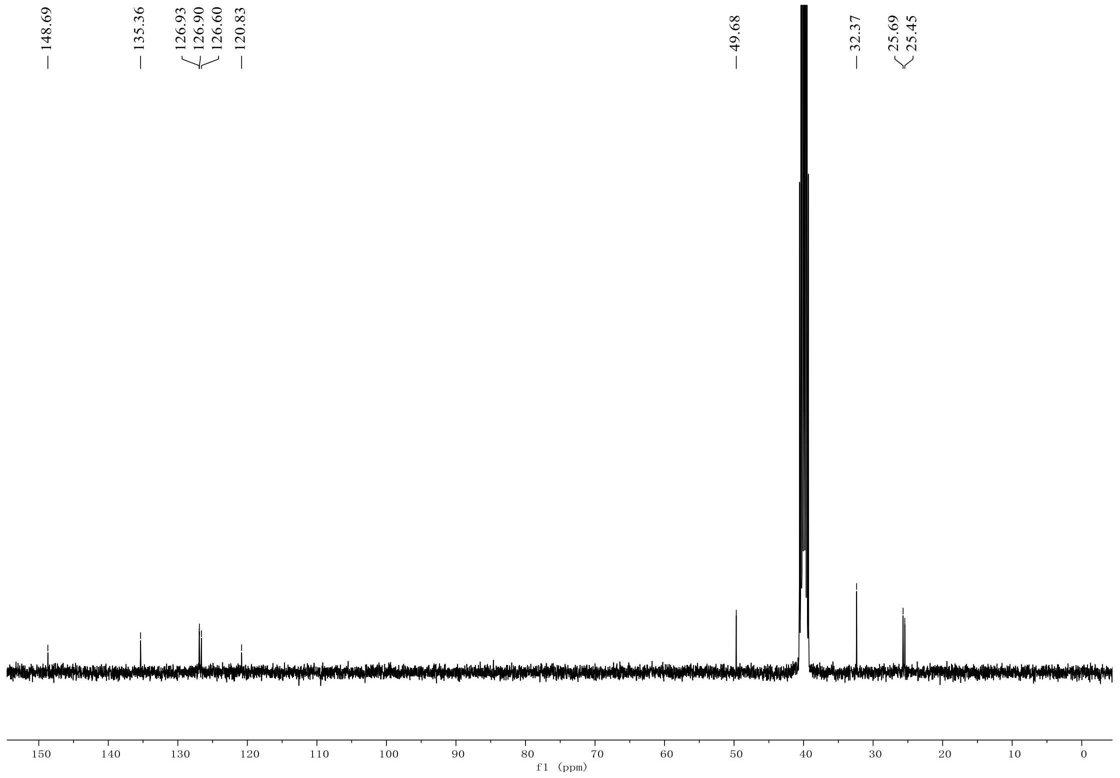

^{13}C NMR of compound 8

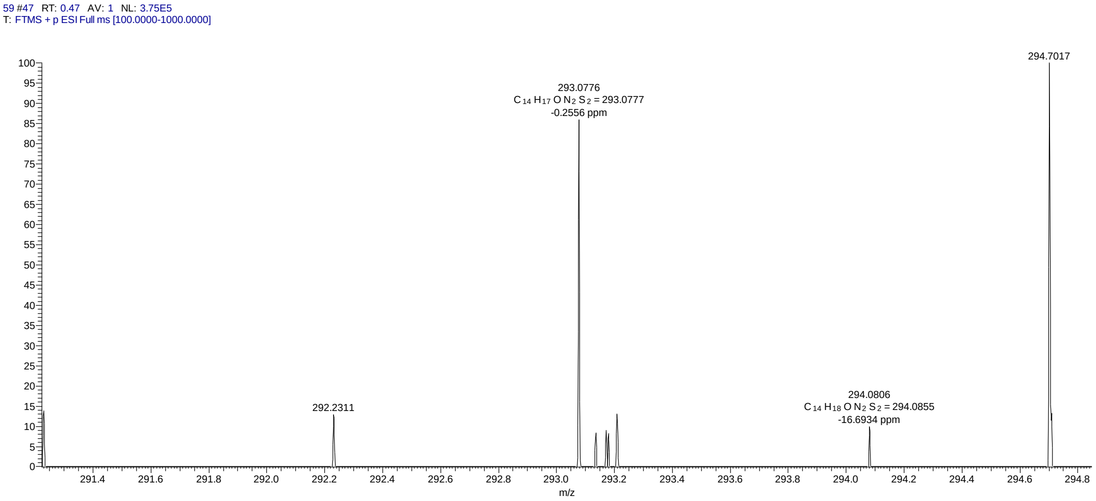

HRMS of compound 8 [M+1]

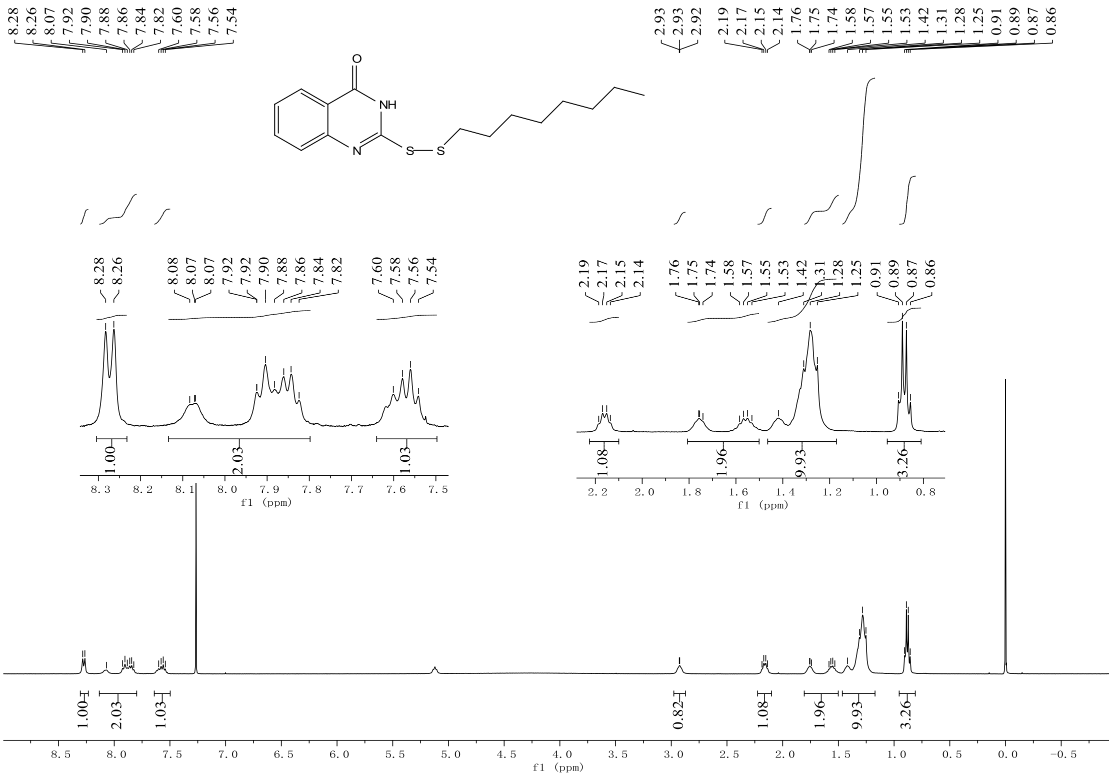

^{1}H NMR of compound 9

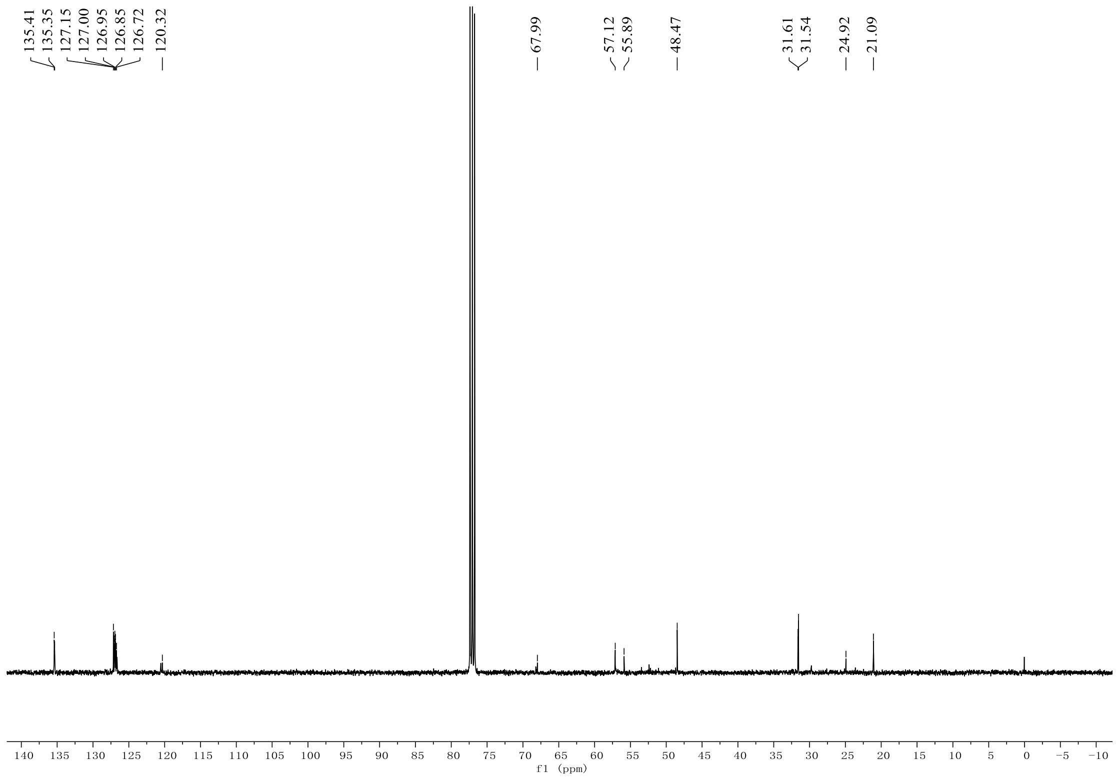

^{13}C NMR of compound 9

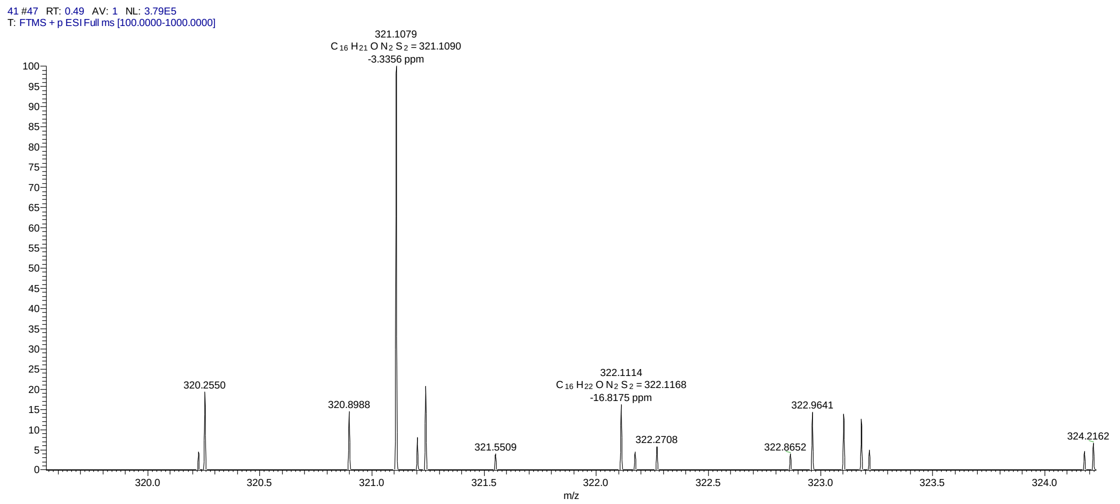

HRMS of compound 9 [M−1]

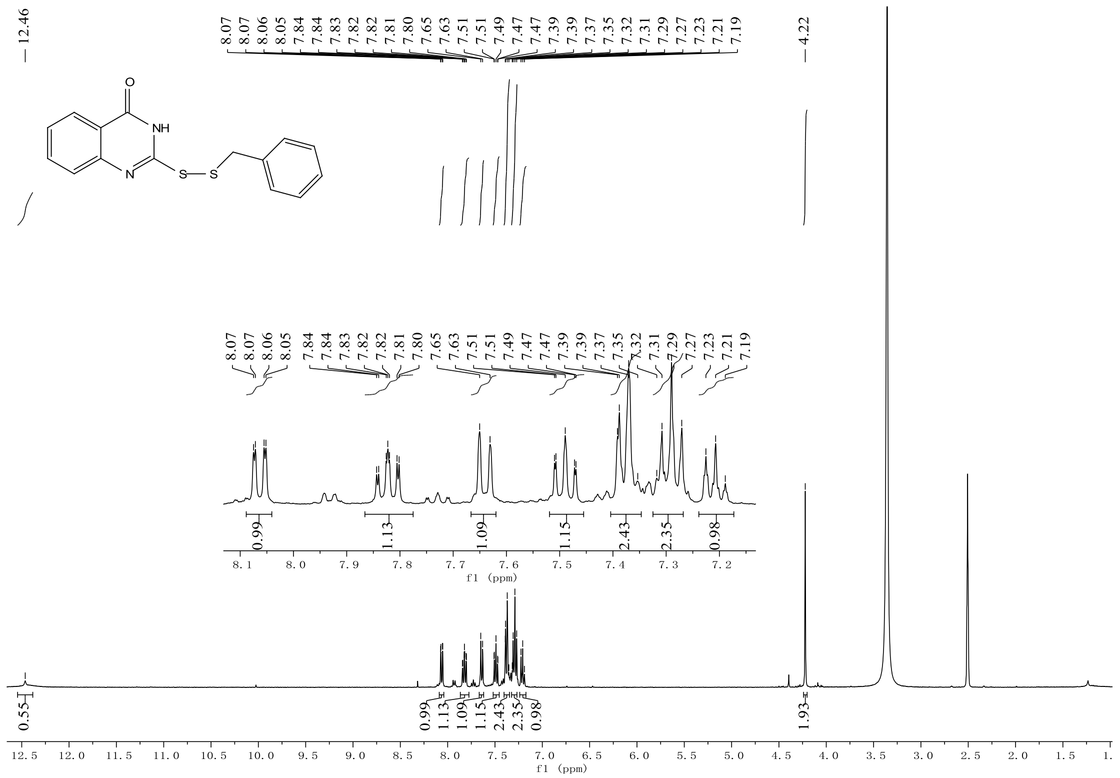

^{1}H NMR of compound 10

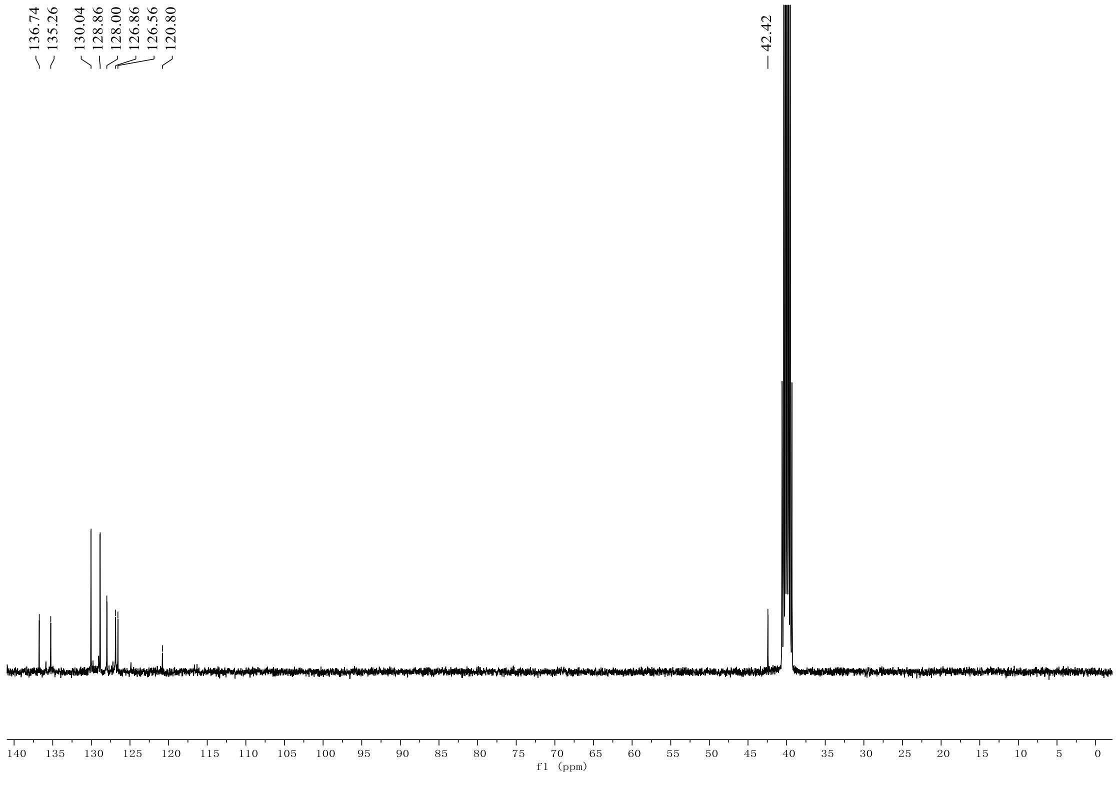

^{13}C NMR of compound 10

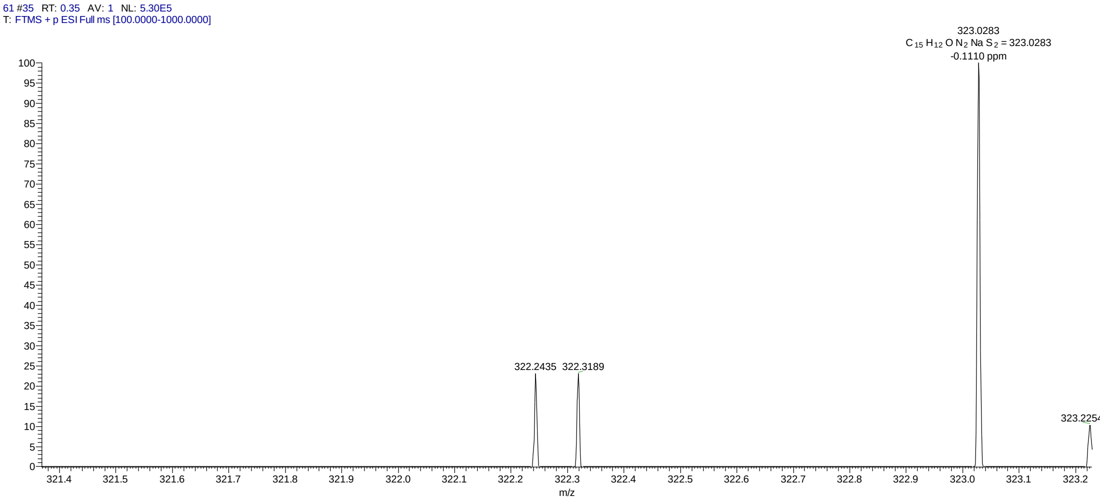

HRMS of compound 10 [M+Na]

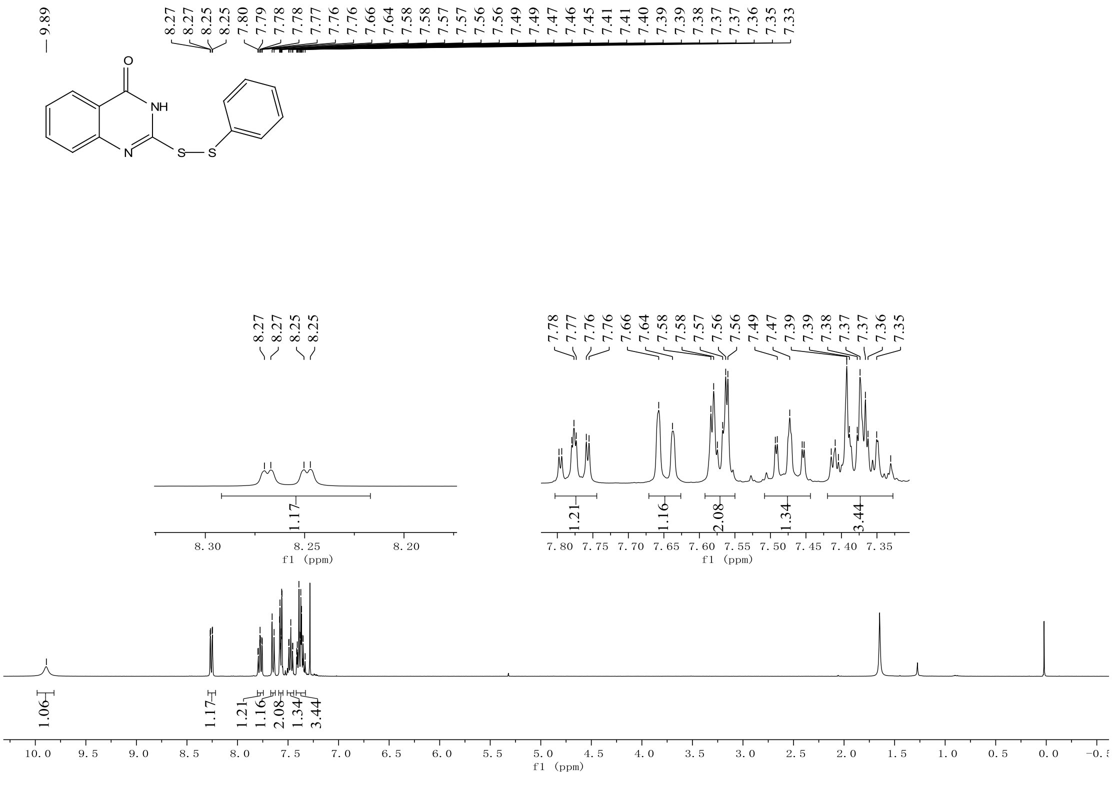

^{1}H NMR of compound 11

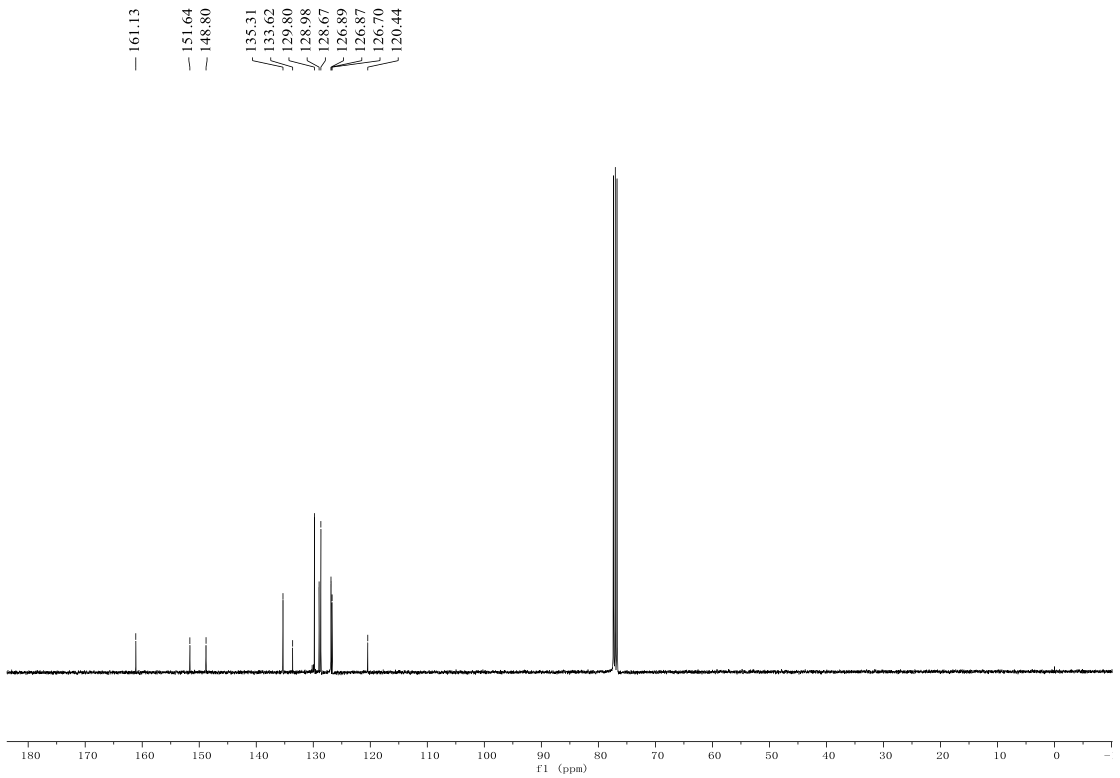

^{13}C NMR of compound 11

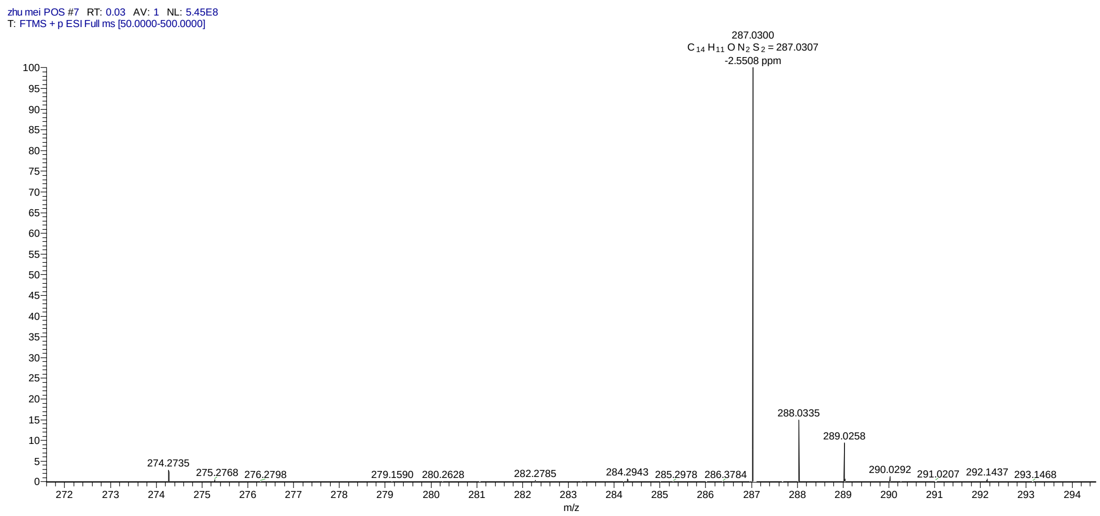

HRMS of compound 11 [M+1]

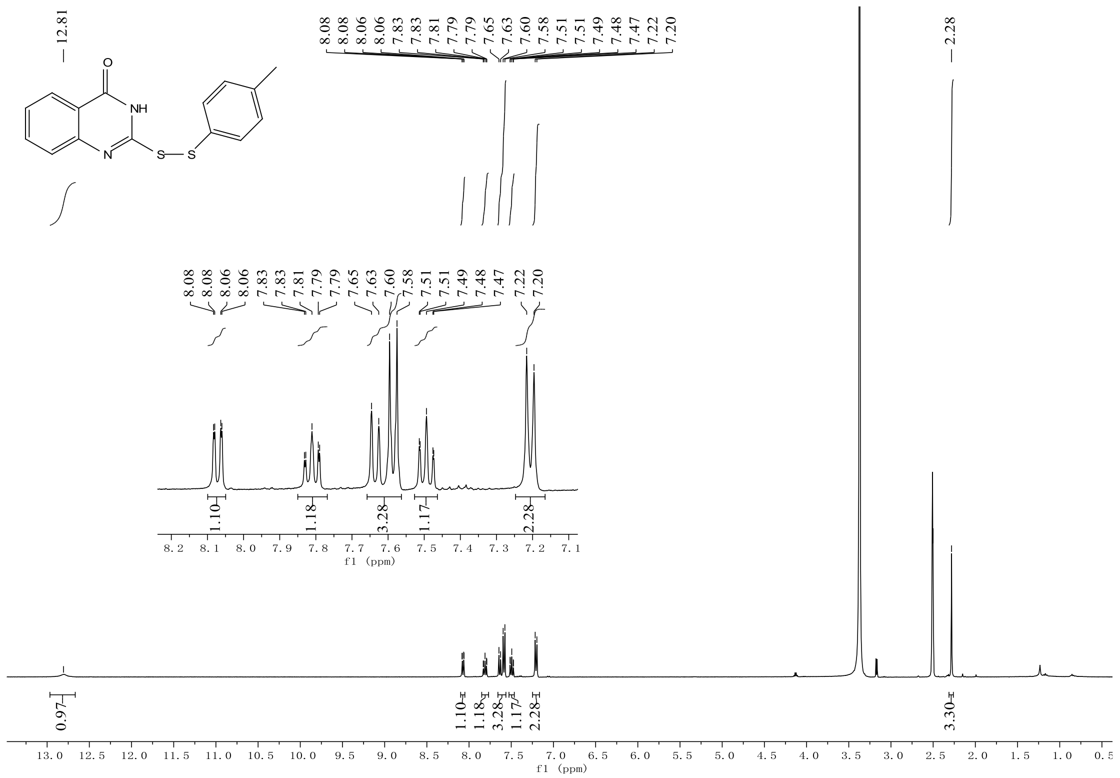

^{1}H NMR of compound 12

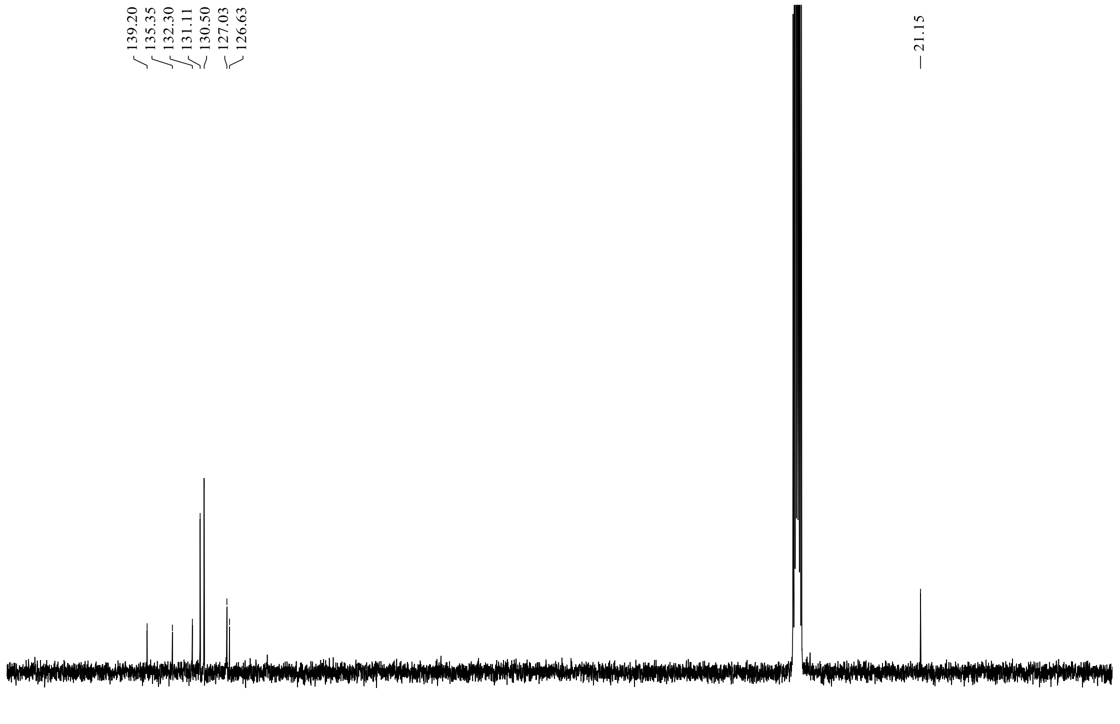

^{13}C NMR of compound 12

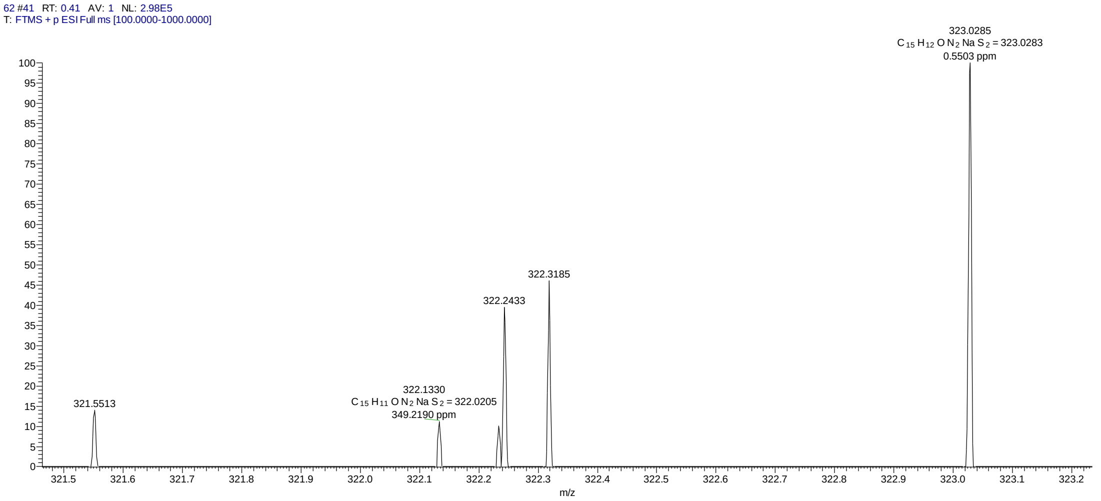

HRMS of compound 12 [M+Na]

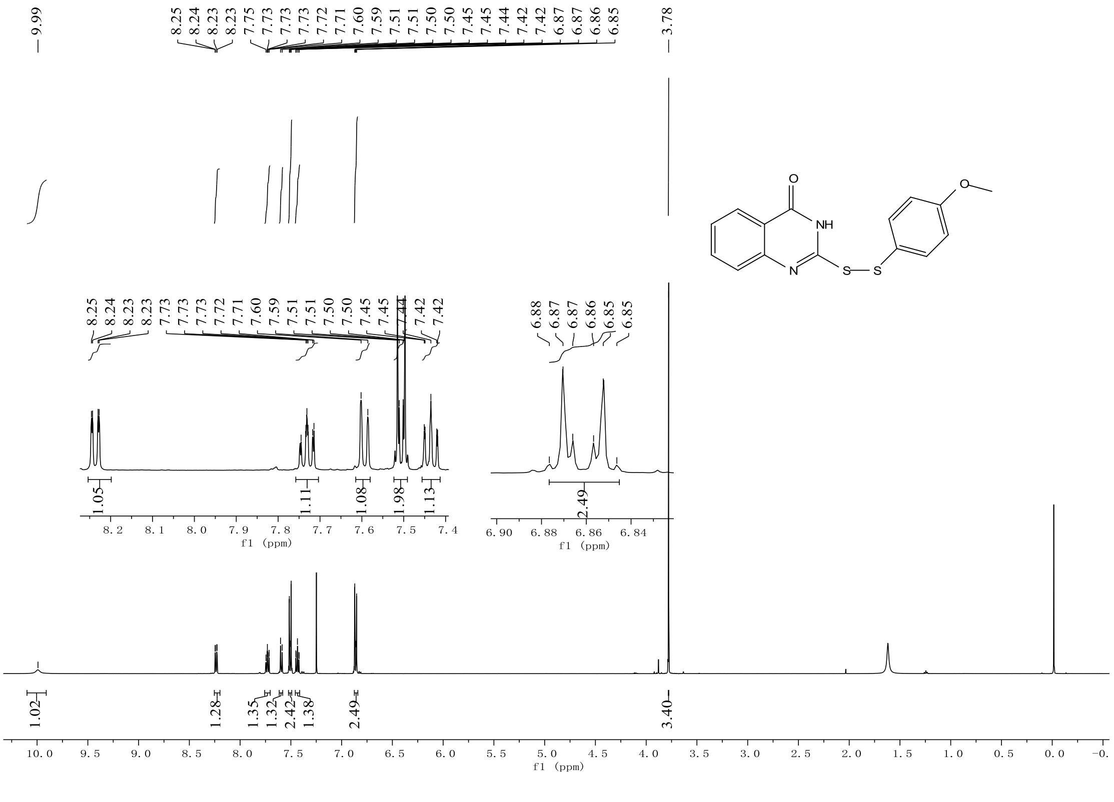

^{1}H NMR of compound 13

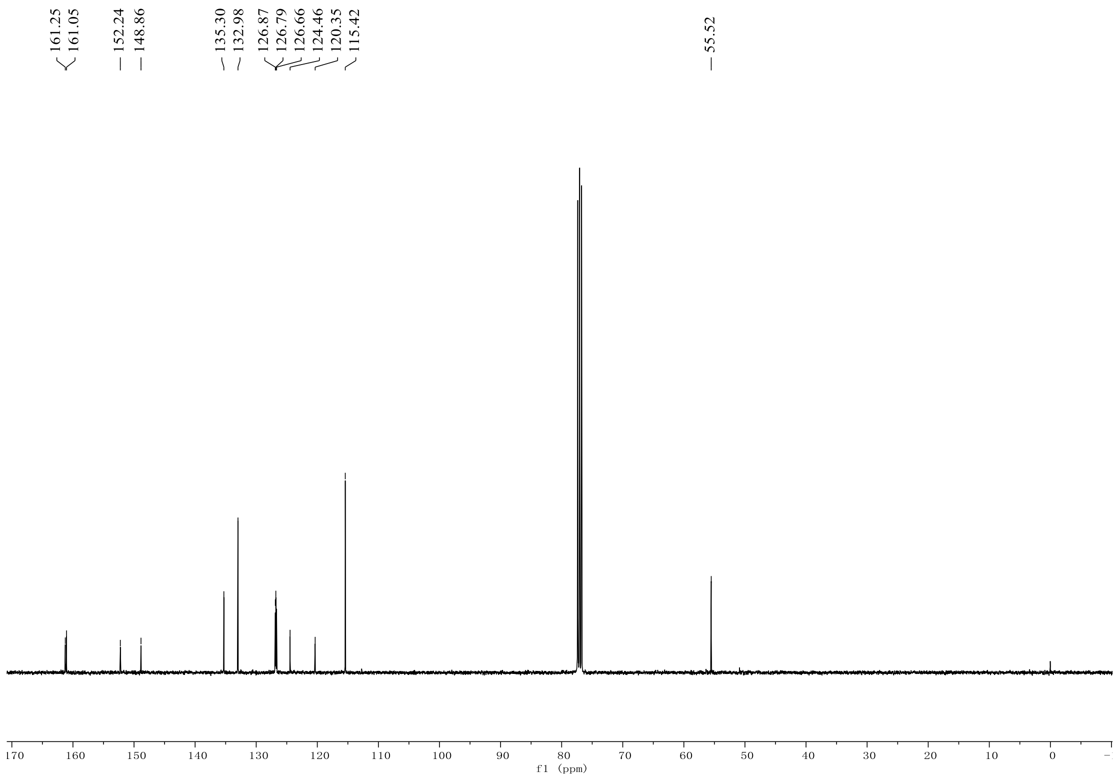

^{13}C NMR of compound 13

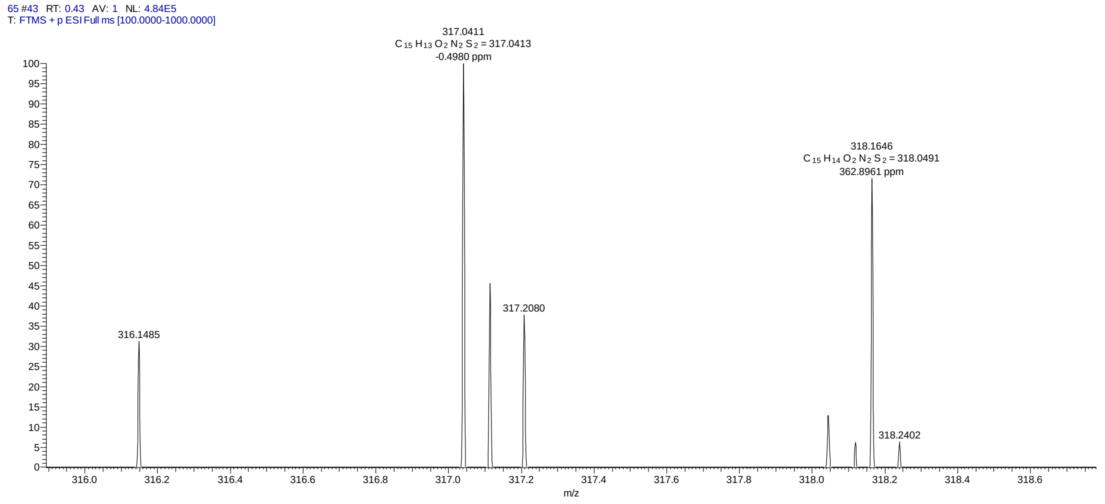

HRMS of compound 13 [M+1]

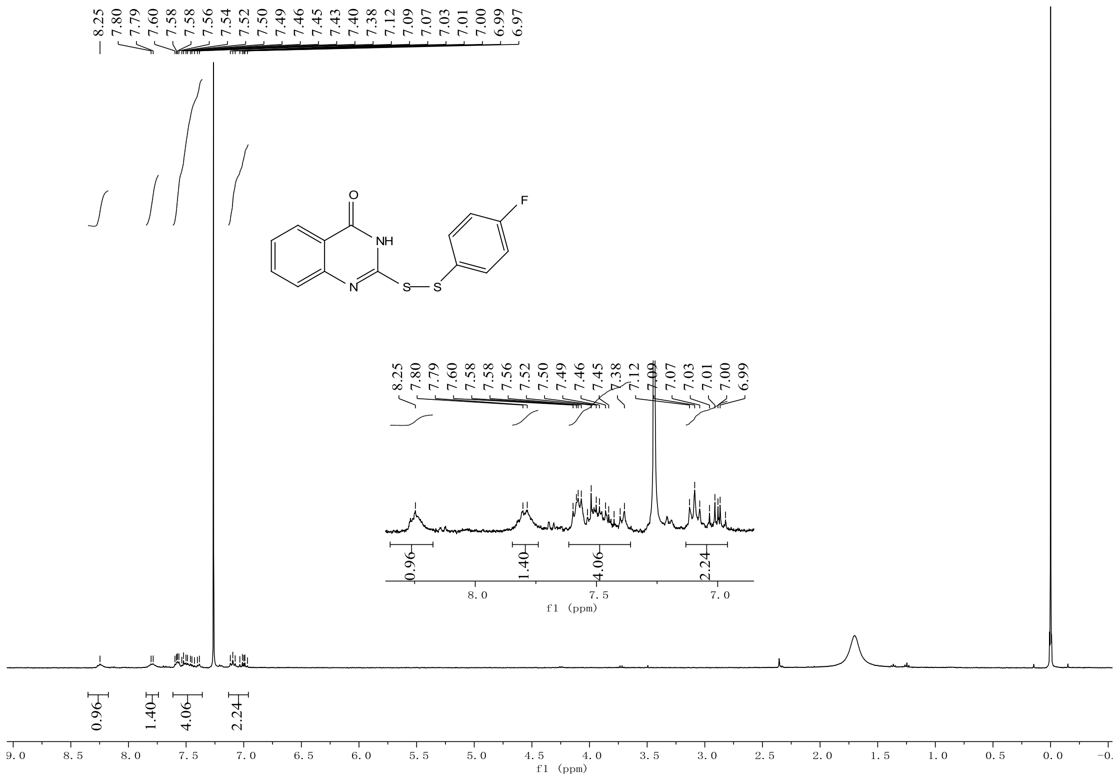

¹H NMR of compound 14

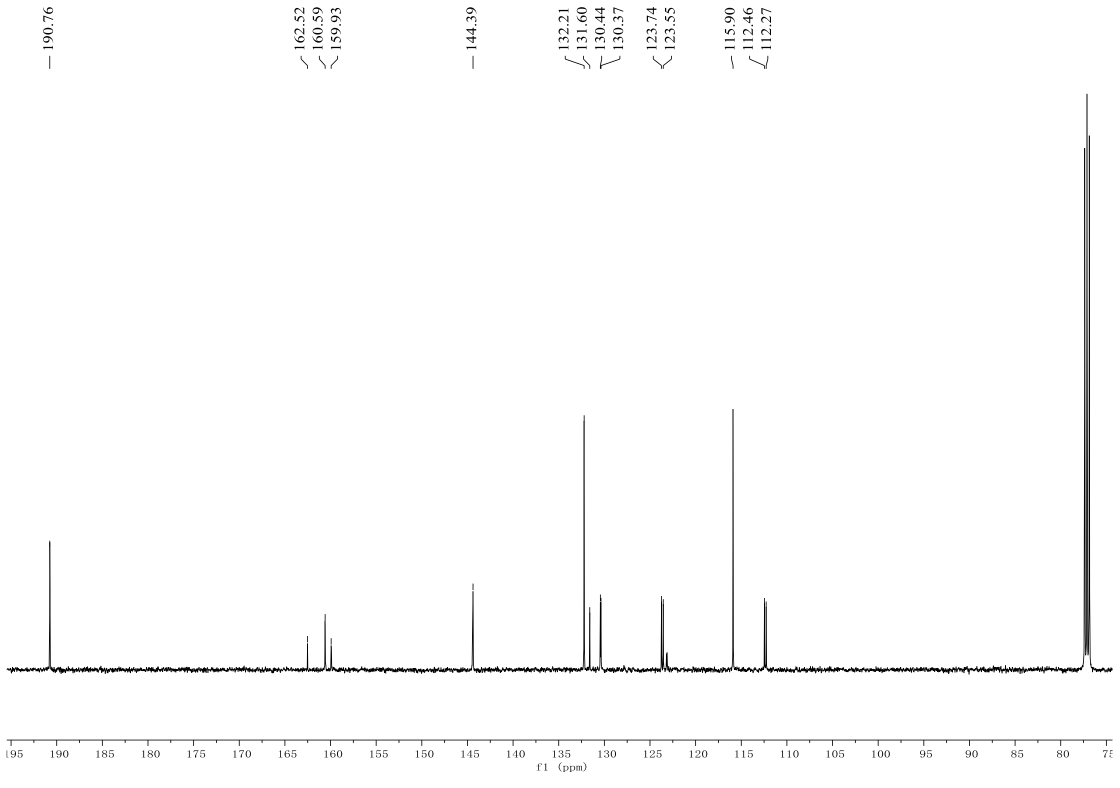

¹³C NMR of compound 14

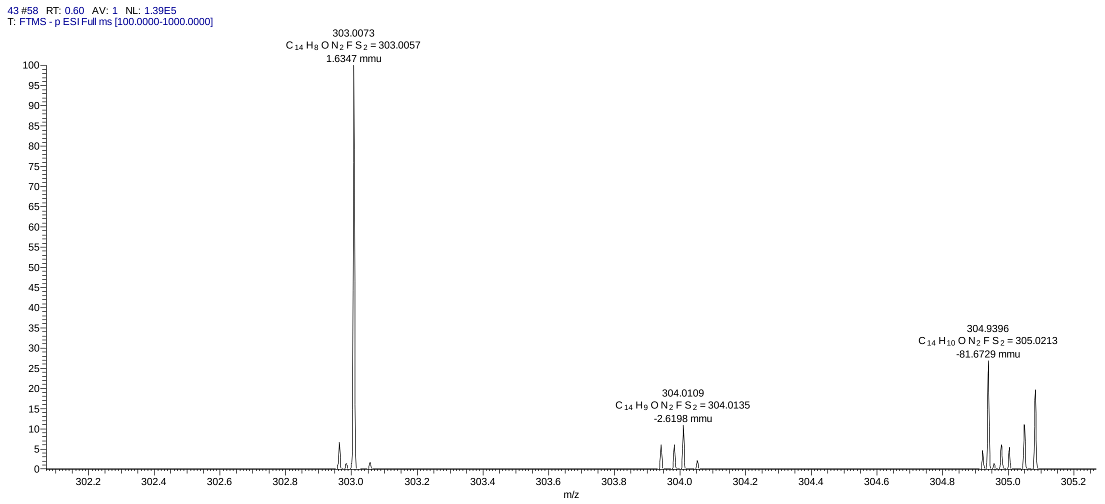

HRMS of compound 14 [M−1]

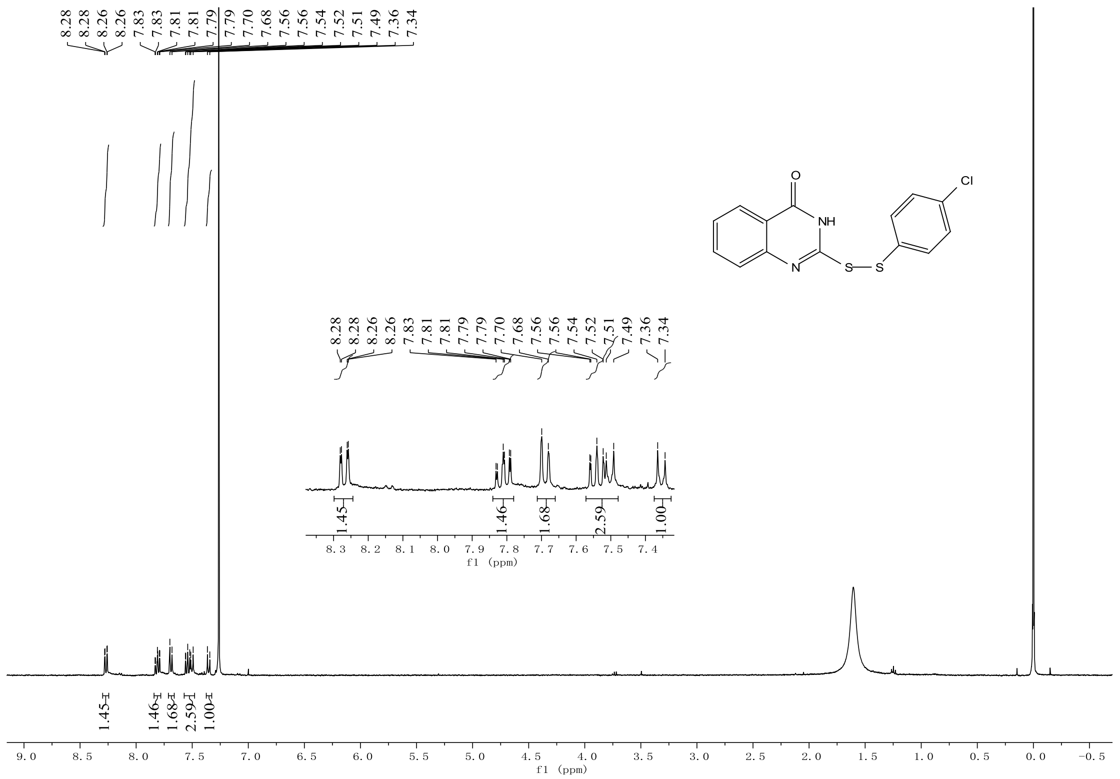

^{1}H NMR of compound 15

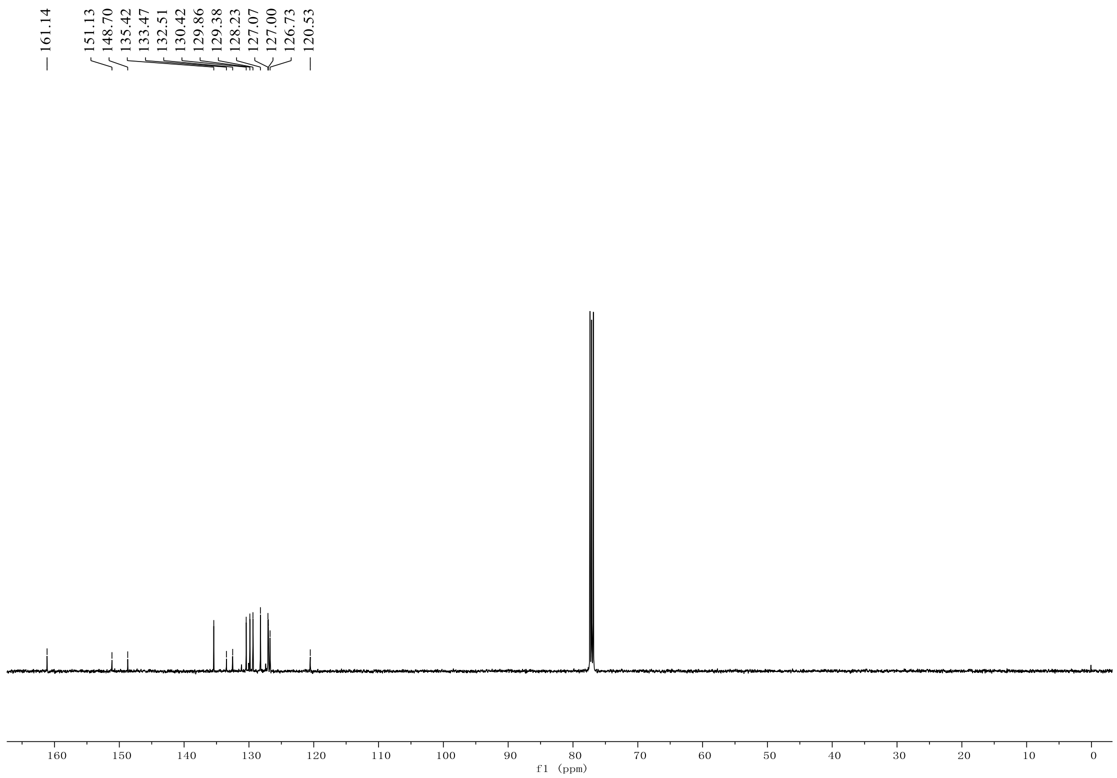

^{13}C NMR of compound 15

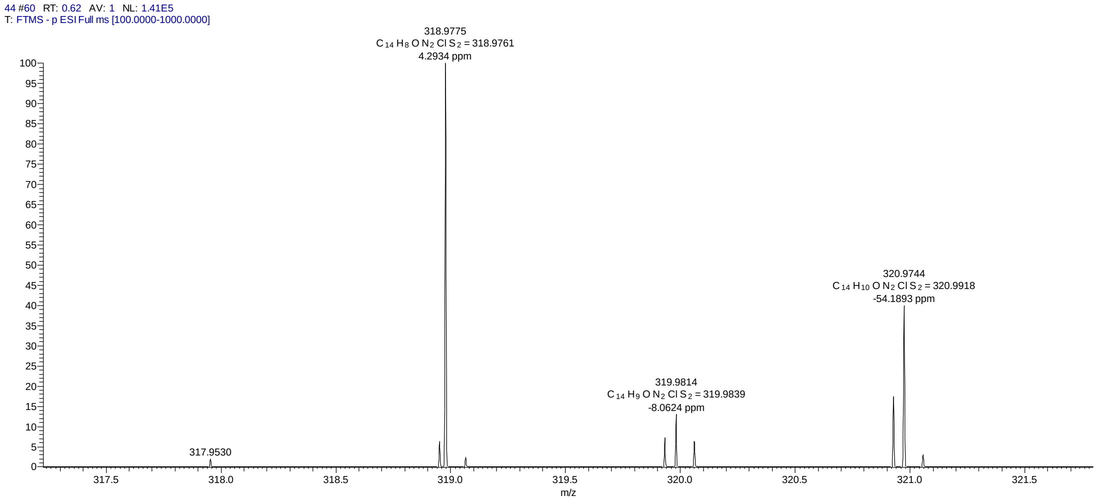

HRMS of compound 15 [M−1]

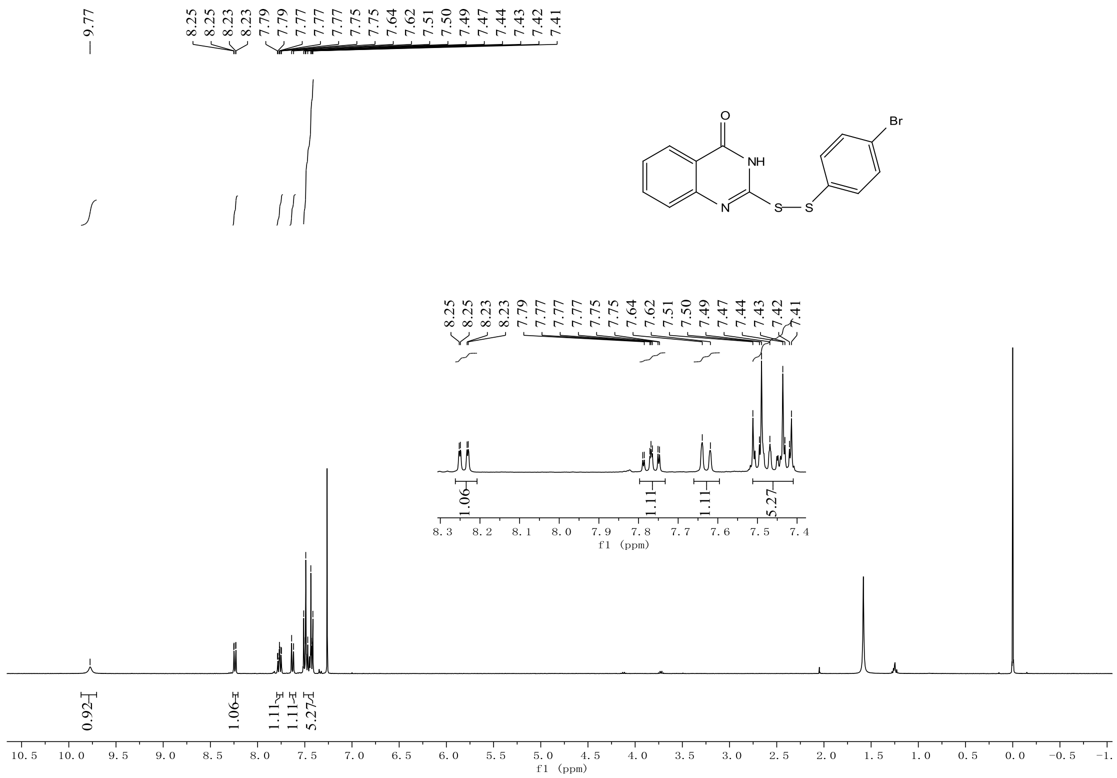

^{1}H NMR of compound 16

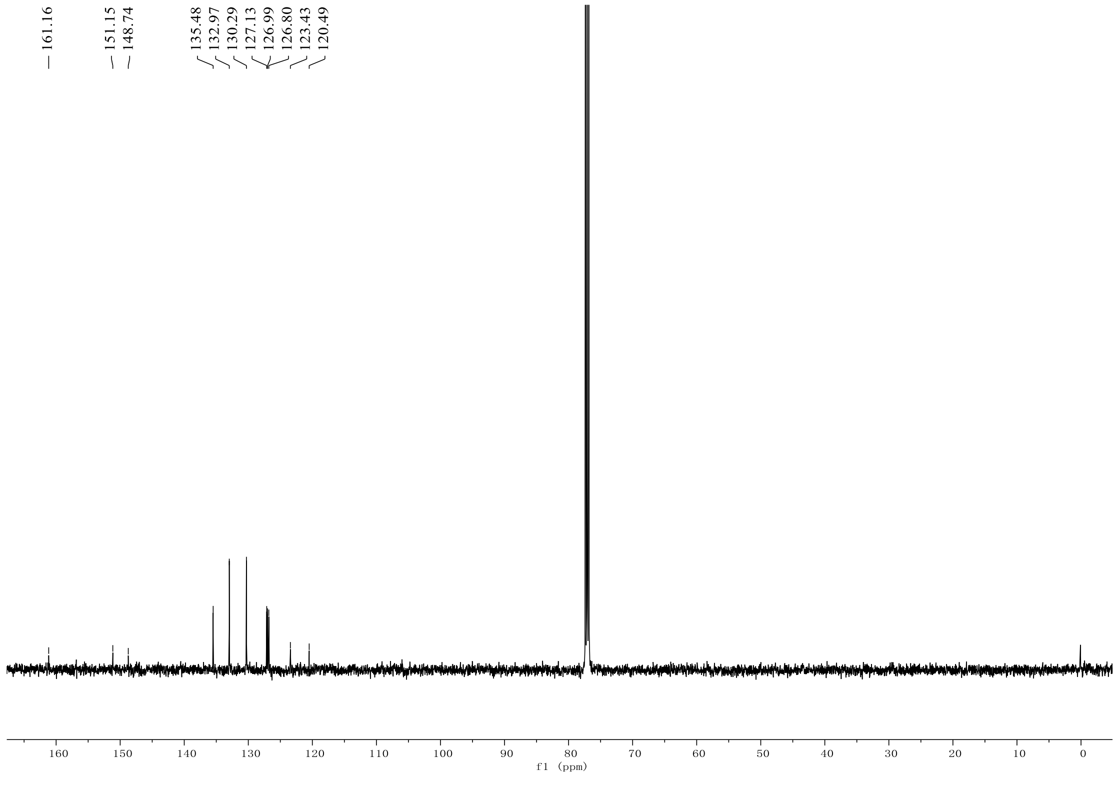

^{13}C NMR of compound 16

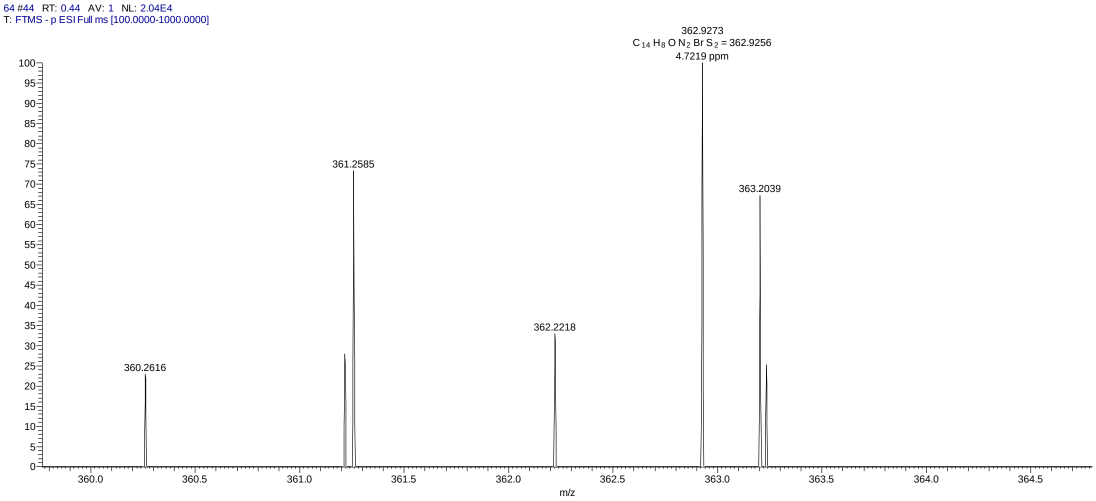

HRMS of compound 16 [M−1]

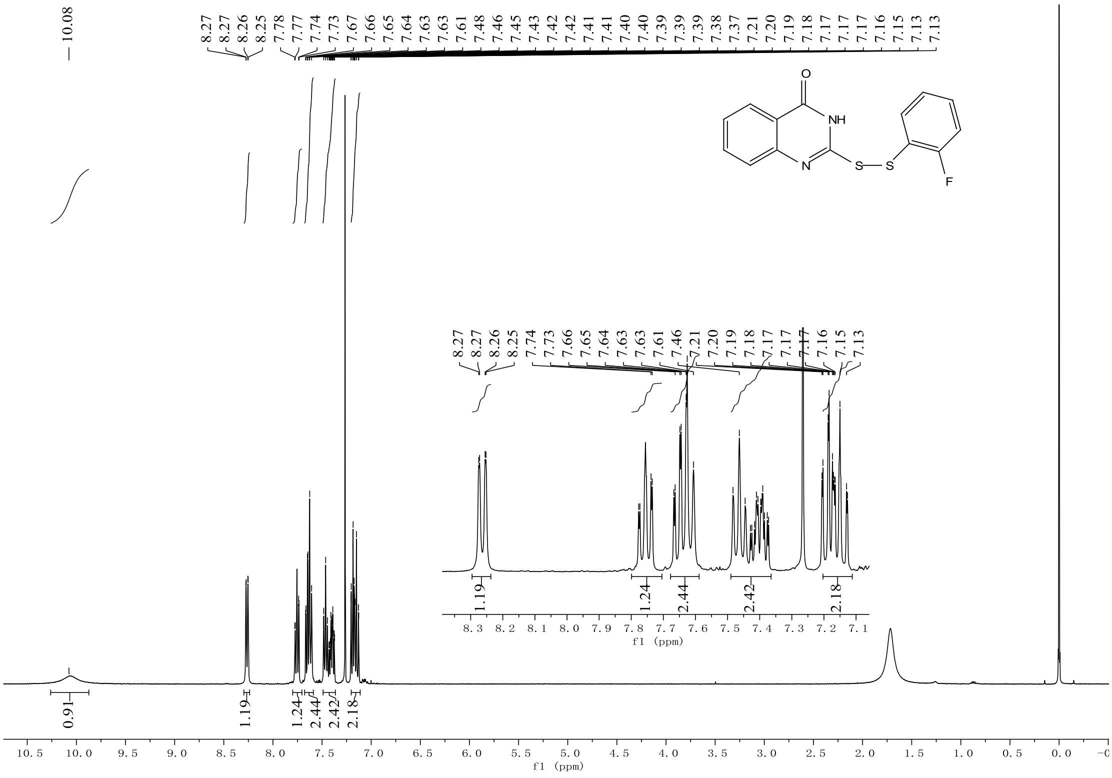

^{1}H NMR of compound 17

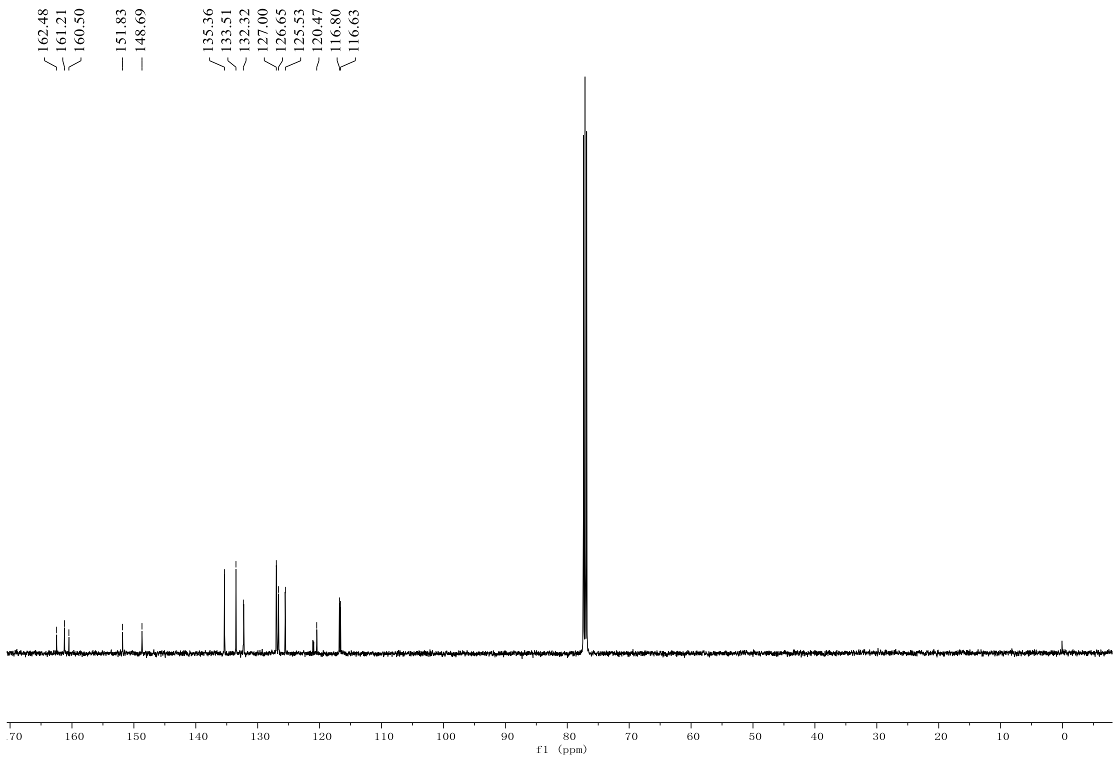

^{13}C NMR of compound 17

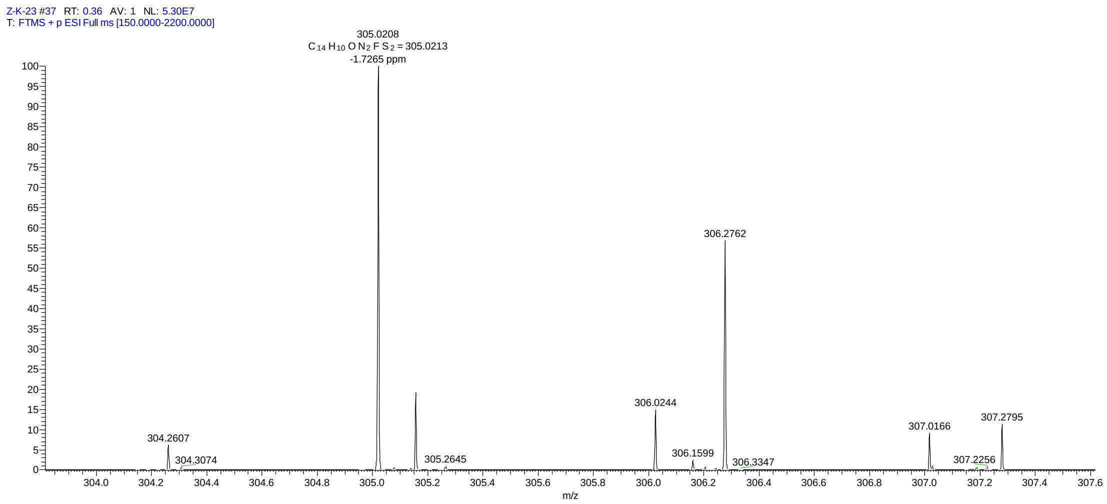

HRMS of compound 17 [M+1]

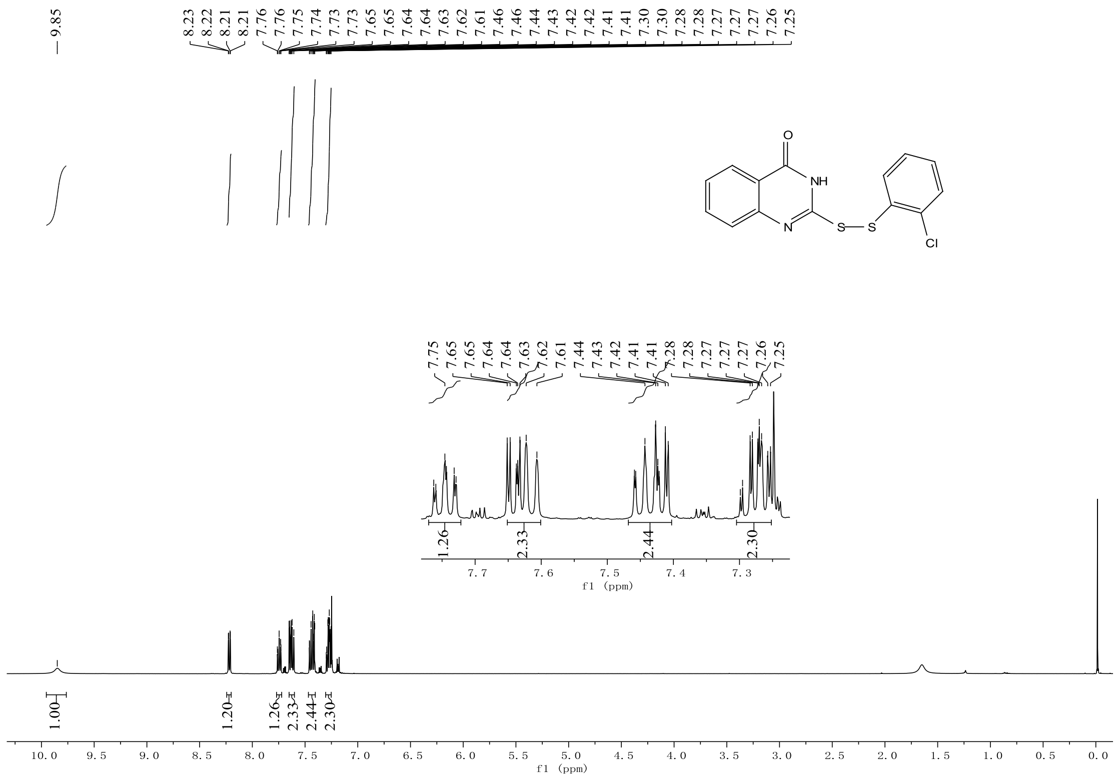

^{1}H NMR of compound 18

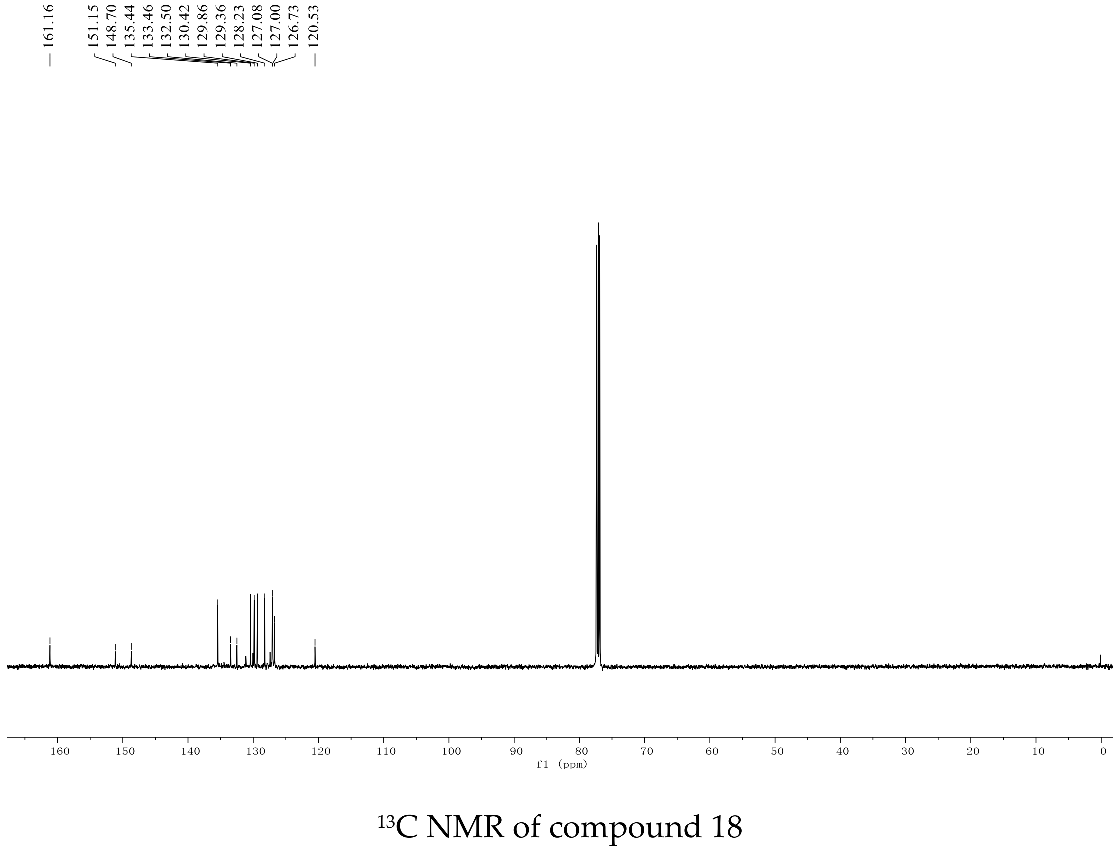

^{13}C NMR of compound 18

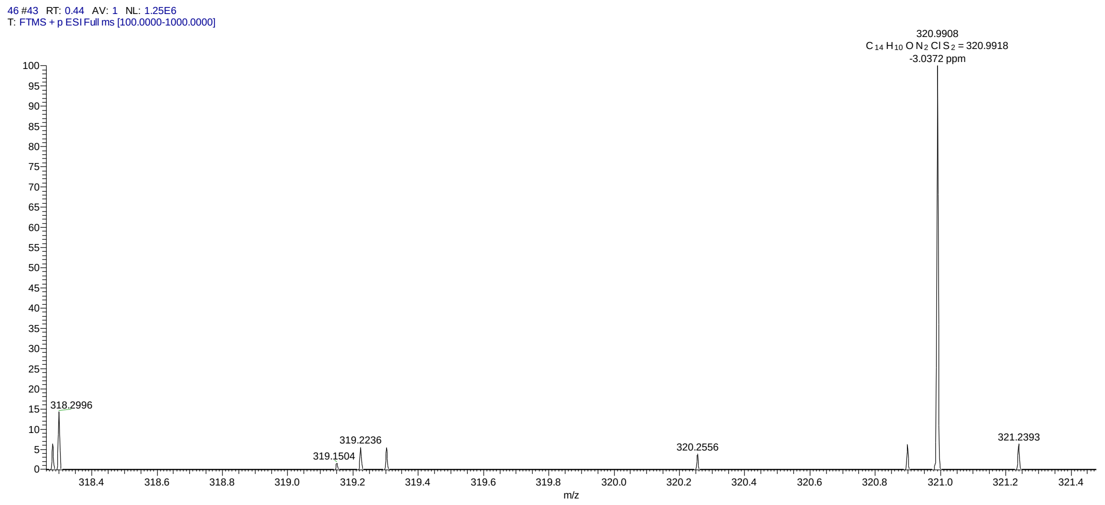

HRMS of compound 18 [M+1]

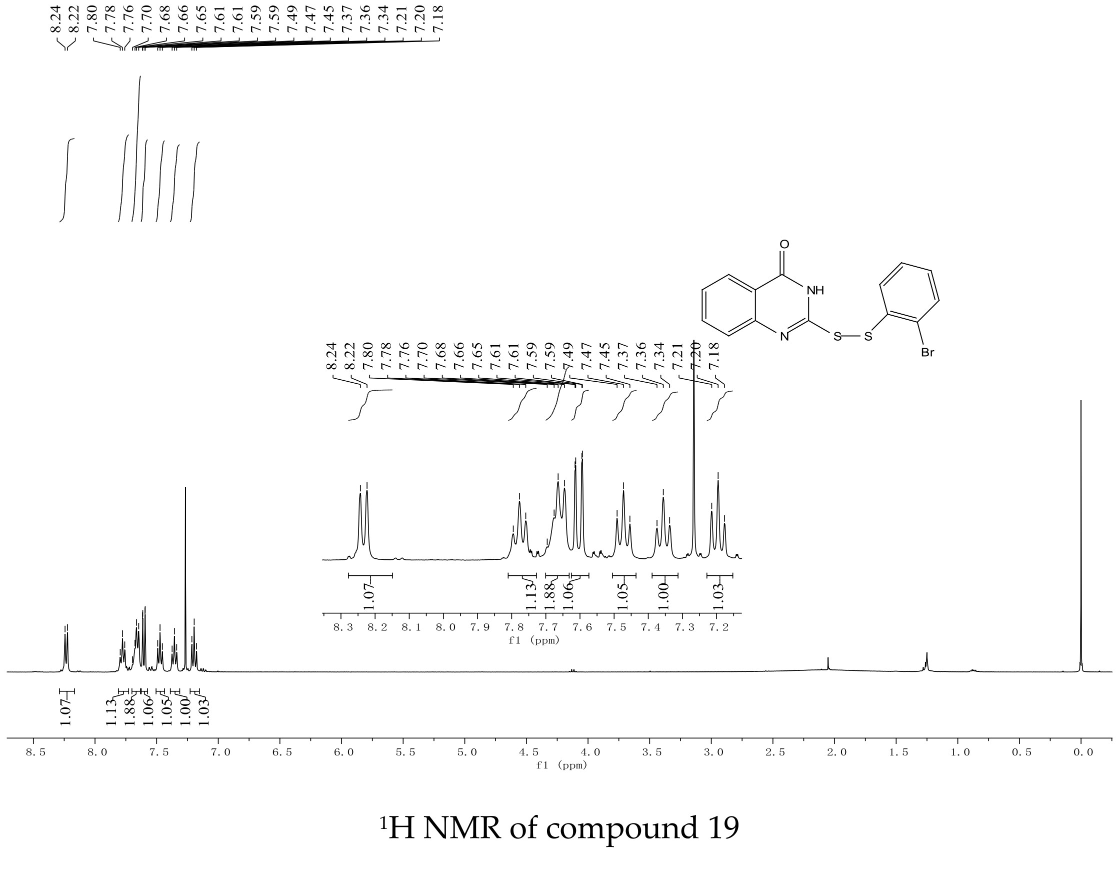

^{1}H NMR of compound 19

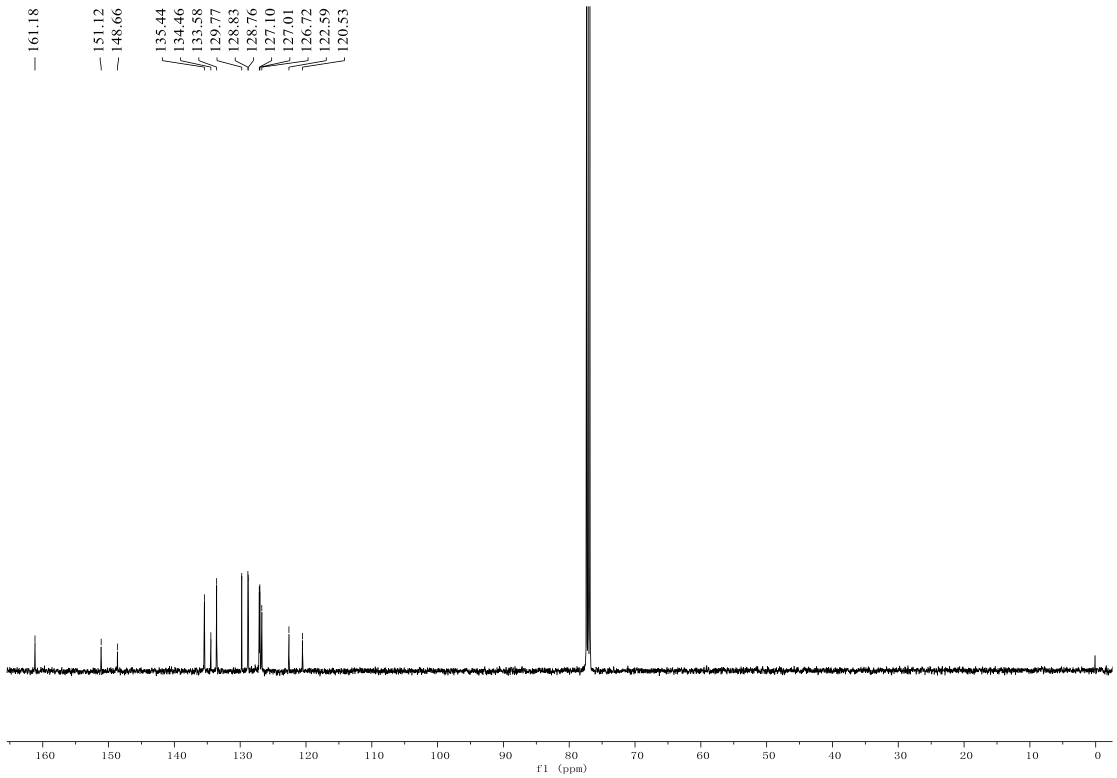

^{13}C NMR of compound 19

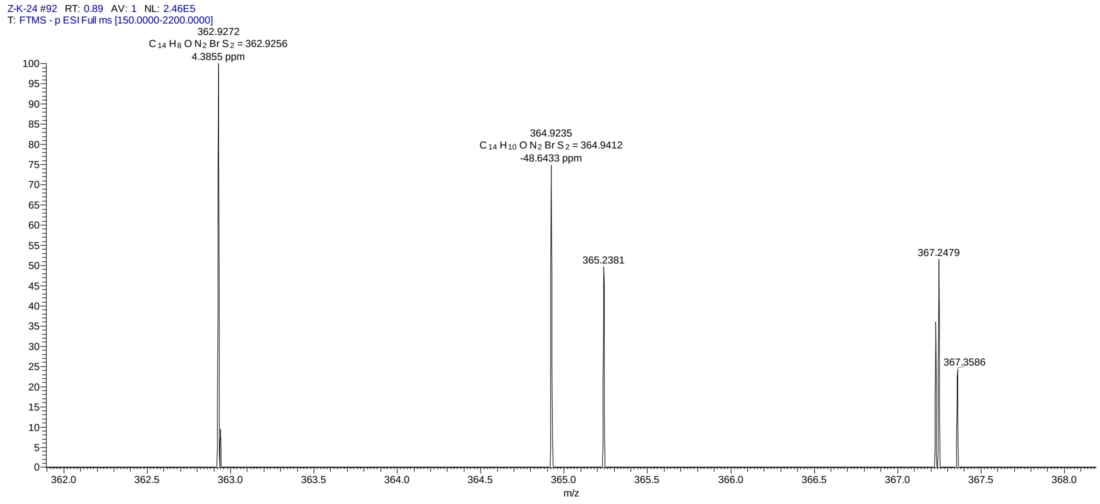

HRMS of compound 19 [M−1]

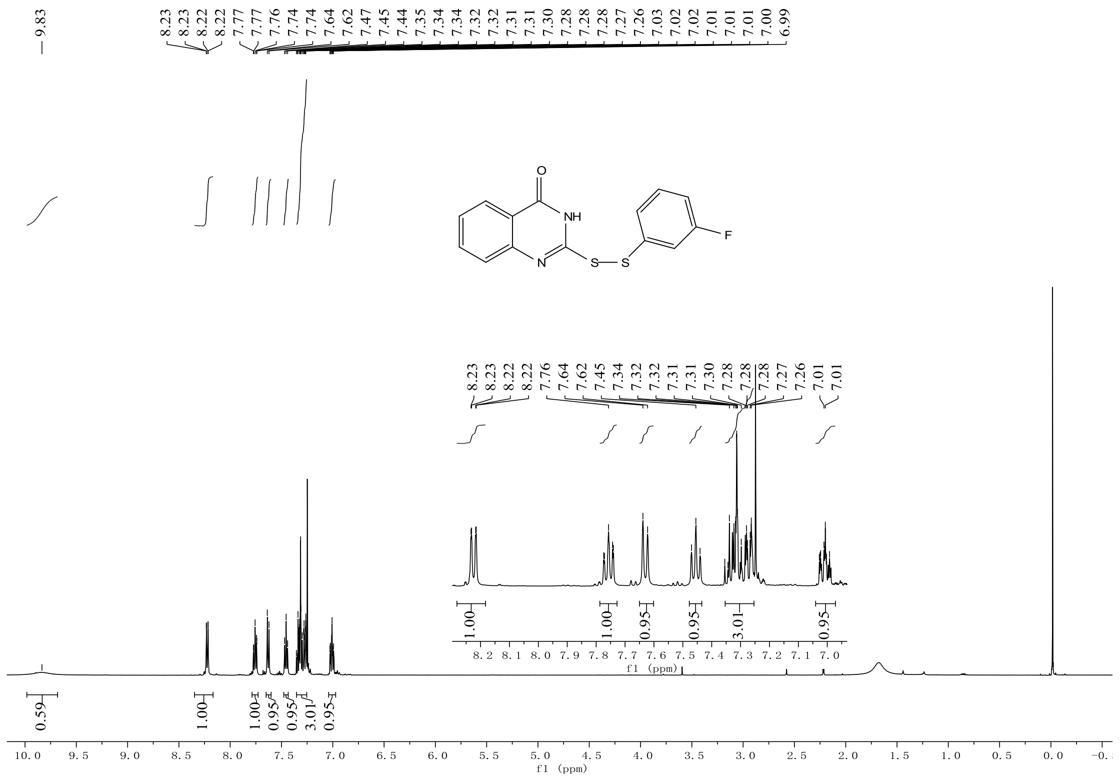

^{1}H NMR of compound 20

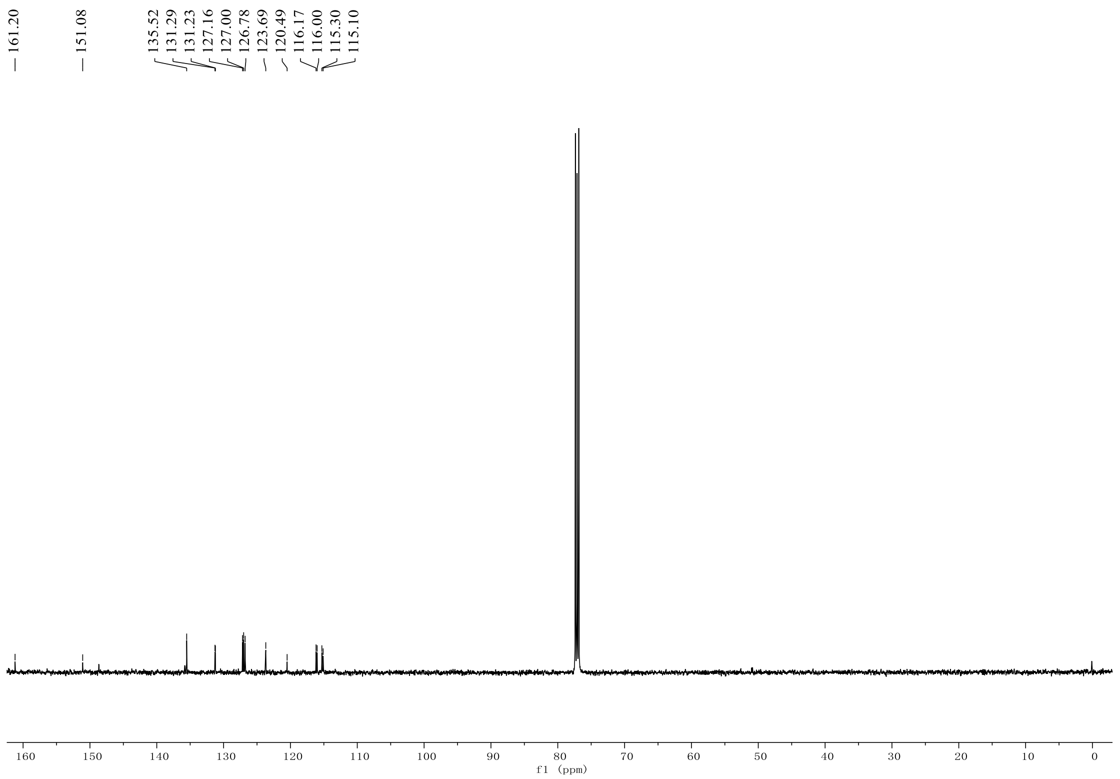

^{13}C NMR of compound 20

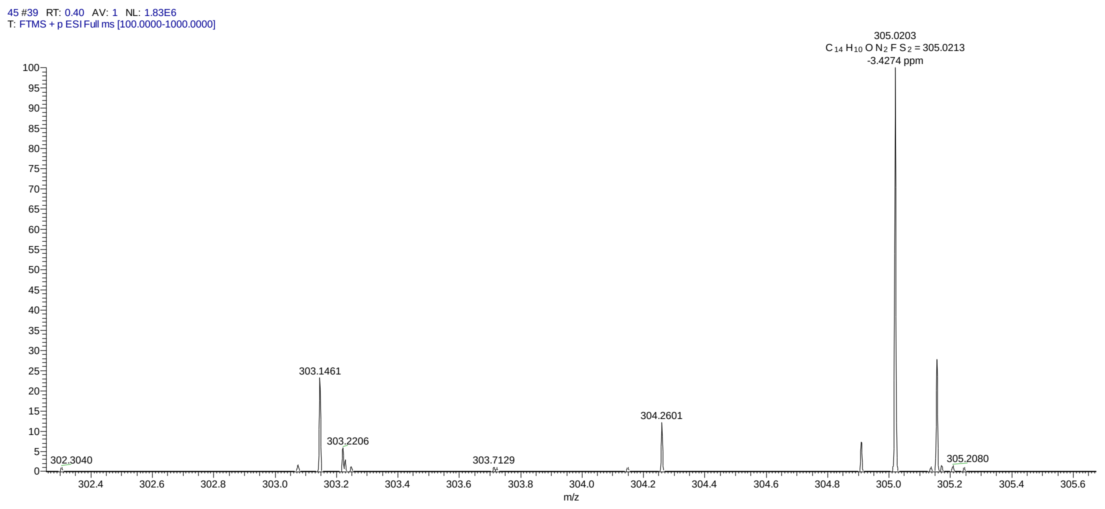

HRMS of compound 20 [M+1]

Table S1. The concentrations of the target compounds involved in the regression equation and its corresponding inhibition rates.

Compound	concentration (μg/mL)	Inhibition rate	X (log concentration)	Y (5+NORMSINV (Inhibition rate))	regression equation
1	6.25	97.37%	0.79588	6.94	y = 4.64x + 3.09
	3.125	53.46%	0.49485	5.09	
	1.5625	19.63%	0.19382	4.14	
2	12.5	97.50%	1.09691	6.96	y = 4.78x + 1.48
	6.25	42.81%	0.79588	4.82	
	3.125	17.91%	0.49485	4.08	
3	12.5	96.44%	1.09691	6.80	y = 4.60x + 1.51
	6.25	36.80%	0.79588	4.66	
	3.125	16.79%	0.49485	4.04	
4	25	99.32%	1.39794	7.47	y = 4.74x + 0.64
	12.5	67.05%	1.09691	5.44	
	6.25	34.98%	0.79588	4.61	
5	100	97.10%	2	6.90	y = 3.85x - 1.03
	50	53.19%	1.69897	5.08	
	25	33.55%	1.39794	4.58	
10	12.5	98.73%	1.09691	7.24	y = 4.39x + 2.17
	6.25	56.50%	0.79588	5.16	
	3.125	34.21%	0.49485	4.59	
11	6.25	80.76%	0.79588	5.87	y = 2.19x + 4.07
	3.125	50.97%	0.49485	5.02	
	1.5625	32.73%	0.19382	4.55	
20	50	88.89%	1.69897	6.22	y = 1.73x + 3.10
	25	60.38%	1.39794	5.26	
	6.25	33.31%	0.79588	4.57	
TC	100	69.94%	2	5.52	y = 2.56x + 0.31
	50	50.86%	1.69897	5.02	
	25	16.82%	1.39794	4.04	
BM	100	67.67%	2	5.46	y = 2.47x + 0.67
	50	34.82%	1.69897	4.61	
	25	13.96%	1.39794	3.92	

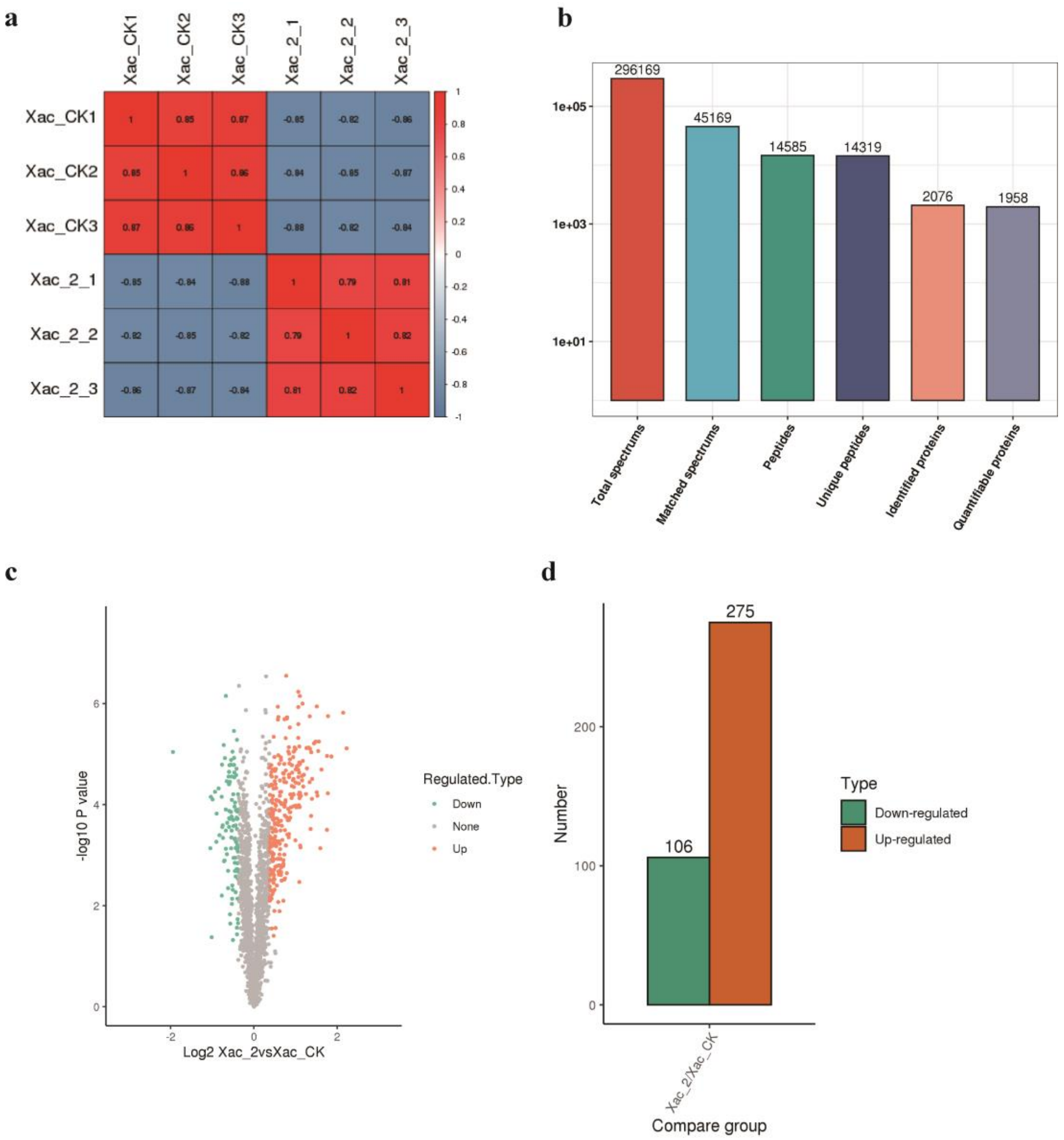

Figure S1. Results of quantitative Proteomics. **a**, Pearson's correlation coefficient; **b**, Overview of protein identification; **c**, The volcano map of differential protein; **d**, Statistical chart of differential protein.

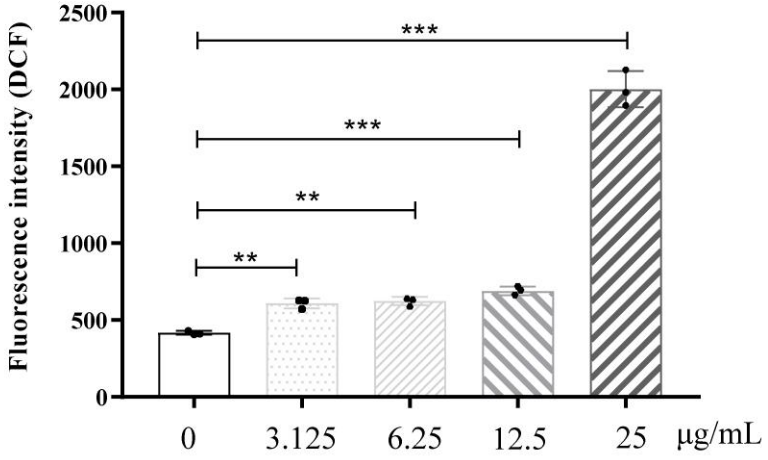

Figure S2. Results of ROS (reactive oxygen species) detection. Reactive Oxygen Species Assay Kit (Beyotime Biotech, China) was used, this sample treatment same as Apoptosis Detection. Centrifuged at 9500 rpm for 5 min, washed with PBS, resuspended with DCFH-DA, washed three times with PBS after incubation for 20 min, and measured by BD FACSCalibur flow cytometry. From the result, the content of ROS has changed slightly as the concentration increased, however, when the drug concentration is increased to 25 μg/mL, the level of ROS has increased dramatically. * for $P<0.05$, ** for $P<0.01$, ***$P < 0.001$, ns, not significant; One-way ANOVA followed by Dunnett's multiple comparisons test was performed using GraphPad Prism.
